# Associating Psychological Factors With Workplace Satisfaction and Position Duration in a Sample of International School Teachers

**DOI:** 10.3389/fpsyg.2020.601554

**Published:** 2021-01-20

**Authors:** Ross C. Hollett, Mark McMahon, Ronald Monson

**Affiliations:** ^1^School of Arts and Humanities, Edith Cowan University, Joondalup, WA, Australia; ^2^OnSoc, onsoc.com, Perth, WA, Australia

**Keywords:** teacher, schools, retention, satisfaction, psychological

## Abstract

To be an effective teacher, a combination of specific professional skills and psychological attributes are required. With increasingly fluid employment conditions, particularly in the international context, recruiters and schools are under considerable pressure to quickly differentiate candidates and make successful placements, which involves more than just determining if a candidate holds an appropriate qualification. Therefore, the aim of this cross-sectional study was to measure theoretically and empirically valuable psychological attributes in an international sample of schoolteachers to determine the most valuable correlates of satisfaction and position duration. An international sample (*N* = 335) of elementary, middle and high school teachers completed an online survey to capture their workplace satisfaction, position duration and measure 15 psychological attributes using validated instruments. Linear associations were estimated using hierarchical regression with this analysis complemented and compared with follow-up non-linear neural network models. Using regression, lower agreeableness (less people-oriented) emerged as the strongest correlate of longer position duration throughout the cohort. In elementary school teachers, lower impulsivity and higher organizational commitment emerged as the strongest correlates of longer position duration. In high school teachers, better stress tolerance and higher organizational commitment emerged as the strongest correlates of longer position duration. Using neural networks to suggest predictive models, low levels of neuroticism and impulsivity were the strongest predictors of longer position duration in elementary school teachers. High stress tolerance also predicted high work satisfaction in elementary teachers, whereas it was lower impulsivity that most strongly predicted higher work satisfaction in high school teachers. Innovation tendencies, perhaps surprisingly, appeared as a consistent predictor of lower levels of workplace satisfaction across teaching levels. Honesty-humility also emerged as a predictor of shorter position duration, particularly for primary/elementary teachers scoring above the mean. Taken together the results suggest an interesting balancing act that needs to be struck between hiring people-oriented and innovative teachers who may be more effective and adaptable but also at greater risk of changing position, possibly due to an increased interest and ability to transition into new social environments.

## Introduction

Teachers’ traditional “qualifications” arguably embody a certain, subject-specific competency but convey less directly the psychological attributes often critical for success in a teaching role. For example, while Australian undergraduate and postgraduate teaching qualifications certify Australian secondary school teachers’ pedagogical and disciplinary knowledge, they don’t necessarily convey potentially valuable psychological strengths such as personality profile, social inclinations, organizational values, and individual motivations. One of the challenges associated with increased internationalization of teaching and a fluid workforce is appropriately placing teachers so they can effectively integrate into their role and workplace. Given that schools, internationally, are facing teacher turnover rates between 5–14%, and retention is recognized as a critical factor in the functioning of an organization (Odland and Ruzicka, 2009; Australian Institute for Teaching and School Leadership, 2016; Hom et al., 2017), schools are under considerable pressure to make effective recruitment decisions which offer both short term efficiency and long term stability (Sutcher et al., 2016; Ellis et al., 2017). Concerns regarding high turnover rates in the teaching profession are not new and Ingersoll (2001) has argued that school staffing problems are disruptive to the school community and performance. Premature turnover can also be financially costly and time consuming (Allen et al., 2010; Hancock et al., 2011). Therefore, the task of matching a teacher to an appropriate workplace continues to be an important one. One challenge faced by recruiters and school principals is differentiating candidates effectively, particularly when they hold similar qualifications. International placement adds further challenges because the workplaces may involve unfamiliar cultures and customs, requiring aptitudes such as resilience and adaptability, as well as social and emotional competence (Joslin, 2002; Savva, 2015). That is, the burden of turnover in international schools is often higher because of relocation, local training, and supporting family living costs (Hayden and Thompson, 2008; Savva, 2015; Miguel Dos Santos, 2019). Therefore, protecting the international teaching workforce and enhancing stability in international schools through informed recruitment is a particularly valuable objective. This can be achieved by better understanding the association between individual psychological factors and professional outcomes in teachers placed across international teaching contexts.

Research examining the factors that predict organizational outcomes has long supported the value of measuring individual psychological attributes (Judge et al., 2001, 2002). Indeed, in a recent meta-analysis exploring 57 predictors of turnover, individual attributes such as personality and motivational attitudes were identified as valuable predictors (Rubenstein et al., 2018). Consequently, the aim of this study was to review the literature to identify and then measure theoretically and empirically relevant psychological attributes in a sample of internationally placed educators to determine which attributes correlate with, and potentially predict, placement success.

## Literature Review

### Markers of a Successful Teacher Placement

High satisfaction and retention are important indicators of when people are well matched to a position and an organization (Hom and Kinicki, 2001; Ingersoll, 2001; Bogler and Nir, 2014). A teacher is more likely to remain in a position and report high satisfaction if they perceive that they are utilizing their knowledge, skills and abilities to perform well (Cable and DeRue, 2002; Bogler and Nir, 2014). Their satisfaction is also dependent on sharing similar fundamental characteristics to other individuals in the organization and the perception that their needs are met by the organization (Carless, 2005; Price, 2011; Ellis et al., 2017; Rubenstein et al., 2018). Evidence also suggests that satisfaction is correlated with performance (Judge et al., 2001; Bowling et al., 2015), organizational citizenship behaviors (Saks Alan, 2006; Ilies et al., 2009; Zeinabadi, 2010) and school effectiveness (Hulpia et al., 2009). Job satisfaction has been assumed to reflect an employee’s appraisal of their work, tasks, supervision and remuneration with regards to the pleasure or positive emotion that it elicits (Locke, 1969; Schleicher et al., 2004; Judge et al., 2017). In the present study, however, we chose to measure *workplace satisfaction*, since all the participants were assumed to be performing the same job (teaching) and it was more important to determine, in this context, whether a teacher found their specific workplace satisfying.

Retention, by contrast, has often been assumed to be a key behavioral marker for organizational commitment (Hom, 2012). However, retention behaviors can also be dependent on economic climates and job markets (Hom, 2012; Hom et al., 2017), which are often precarious in the international schooling sector (Bunnell, 2019). Given that turnover intentions can be predicted by organizational commitment and job satisfaction in some industries (Kim and Kao, 2014; Mathieu et al., 2016), it serves as a valuable indicator of placement success. High turnover in the teaching profession contributes to increased school operating costs and decreased student achievement (Ronfeldt et al., 2013; Hanushek et al., 2016; Sutcher et al., 2016). Understanding what role, if any, psychological attributes might contribute to teacher retention, particularly in international schools, is important for guiding recruitment decisions that aim to reduce the likelihood of premature turnover. In this study, we used *position duration* as a marker of individual retention because it provides an estimate of the suitability of a person to their role within a school and is assumed to inversely relate to turnover. It was also assumed that all participants were employed in teaching positions in their respective workplaces (rather than other types of position). While retention and satisfaction both represent long term outcomes of a successful placement, several studies have found them to be uncorrelated (Spector and Michaels, 1986; Boswell, 2006; Furnham, 2009; Lizano and Mor Barak, 2015; Boswell et al., 2017). Thus they may *potentially* represent distinct markers of a successful placement in some contexts.

For teachers, the capacity to thrive and remain in their role is likely driven by a number psychological factors. Therefore, the purpose of this study was to identify the psychological attributes that best associate with two markers of placement success; workplace satisfaction and position duration. To achieve this, we first provide an overview of the teaching role, including the likely attributes required to effectively integrate into, and perform, in a school. Secondly, we consulted several theoretical frameworks to support these assumptions and assist in identifying relevant correlates. Finally, we examined the psychometric literature to source brief and freely available instruments for measuring these correlates of satisfaction and position duration.

### Psychological Profiling in Teachers

Teaching can been described as an intellectually, socially, and emotionally challenging profession (Chang, 2009), which involves a diverse range of responsibilities. These responsibilities extend beyond simply delivering curriculum and can include creating learning materials, assessing competence, arranging incursions/excursions, conflict resolution, as well as a host of administrative and governance tasks (Comber and Nixon, 2009; Astrauskaite et al., 2011). Importantly, teachers will typically find themselves working with diverse groups of people (e.g., students, colleagues, and parents) who vary in age, cultural background, and motivation, as well as academic and social competency (Comber and Nixon, 2009). These challenges are particularly inherent for teachers in international schools, because they are faced with greater diversity and mobility amongst students and staff compared to national schools (Hayden and Thompson, 2008; Savva, 2015; Bunnell, 2019). Because international schools operate independently to national education systems and usually adopt a different curriculum, teachers may need to be more self-sufficient or rely heavily on their school management for support (Hayden and Thompson, 2008; Odland and Ruzicka, 2009; Bunnell, 2019). International teachers are also unique in their desire to trade familiarity for novelty and challenge (Bailey, 2015; Savva, 2015). These circumstances can lead to insecurity and uncertainty (Bunnell, 2016). As such, well-developed personal attributes are assumed to be especially valuable for teachers attempting to adapt into these demanding environments. A common understanding amongst researchers is that socio-emotional skills in teachers are imperative for maintaining an optimal classroom environment, low levels of conflict, supportiveness and responsiveness to individual needs (Jennings and Greenberg, 2009; Schonert-Reichl, 2017). We also assume that stress management, resilience, positive organizational, and motivational attitudes are potentially valuable predictors of placement success. The extent to which these psychological factors are associated with satisfaction and position duration in international schoolteachers is not well understood. Therefore, it is valuable to identify and measure the attitudes, attributes and motivations that might contribute to both workplace satisfaction and position duration in teachers. To achieve this, we consulted several theoretical frameworks to determine which psychological attributes might best correlate with workplace satisfaction and position duration in international schoolteachers.

### Theoretical and Empirical Framework

According to the prosocial classroom model (Jennings and Greenberg, 2009), teachers who possess certain social and emotional competencies are more capable of coping with the demands of the profession whilst avoiding burnout. Indeed, meta-analytic evidence has shown that there are robust links between teacher stress, negative emotional responses, coping mechanisms, and personality (Montgomery and Rupp, 2005). Importantly, the Collaborative for Academic, Social, and Emotional Learning (CASEL) have developed an educational framework for social and emotional learning (SEL) which details five broad areas of socio-emotional competence (CASEL, 2020). These areas include self-awareness, social awareness, self-management, relationship skills and responsible decision-making (Zins et al., 2004). Jennings and Greenberg (2009) argue that an absence of these competencies in teachers can lead to professional apathy and eventual turnover, while the presence of these competencies can reinforce teacher enjoyment, efficacy, and commitment to the profession. Because socio-emotional skills are fundamental to teacher performance and are thought to reduce the likelihood of stress and burnout (Jones et al., 2013; Jennings et al., 2017), several training programs have been developed for enhancing these competencies (Jennings and Greenberg, 2009; Jones and Bouffard, 2012; Jones et al., 2013; Jennings et al., 2017; Schonert-Reichl, 2017). While dedicated training is one approach for enhancing teacher competencies, it is also possible to select teachers who naturally exhibit skills and attributes which will support their professional wellbeing and sustainability.

From a socio-emotional perspective, there are several important attributes which are considered valuable for performing and enduring in a teaching role. Specifically, to avoid emotional depletion and respond to the needs of others, teachers need to effectively regulate their emotions and be emotionally and cognitively aware of themselves and others (Jones et al., 2013; Jennings et al., 2017). As such, *empathy*, *agreeableness, extraversion*, and *honesty-humility* are traits which may improve the likelihood that a teacher will be able to maintain their wellbeing and longevity in the workplace, through building support networks and avoiding stress. These traits also correspond to the competencies of self-awareness, social awareness and relationship skills defined by CASEL (2020). Specifically empathy is considered a key facilitator of prosocial behavior (Ingoglia et al., 2016) and is typically regarded as multidimensional, comprised of both cognitive and emotional components. Cognitive empathy involves the intellectual understanding of others’ emotions, whereas emotional empathy is the, often visceral, manifestation of an emotional reaction to others’ emotions (Davis, 1983). There is evidence that empathy enhances job satisfaction and reduces turnover in nurses, which is regarded similarly to teaching as a “helping profession” (Williams, 1989; Santo et al., 2013). Empathy also shares similar qualities with agreeableness, although agreeableness can be extended to include selflessness, sympathy, flexibility, and being generally good-natured (McCrae and Costa, 1987). People who are agreeable may also be described as cooperative rather than competitive, with a genuine interest in communion with others (Barrick et al., 2002; Liao and Chuang, 2004). Agreeableness has shown consistently modest (e.g., 0.17) but significant positive correlations with satisfaction in several organizational studies (Mount et al., 2006; Matzler and Renzl, 2007; Zimmerman, 2008; Ilies et al., 2009), which may be explained in part by the effort made by agreeable individuals to maintain positive affect in the workplace through their interactions with colleagues. In contrast, the association between agreeableness and retention is less consistent with some evidence of no association (Burke and Witt, 2004; Furnham, 2009) and some evidence of negative (e.g., −0.25) associations with turnover (Zimmerman, 2008; Swider and Zimmerman, 2010) and intentions to quit (Sarwar et al., 2013). Whether agreeableness correlates with satisfaction in teachers is unclear, however, it did not predict retention in first year United States teachers (Bastian et al., 2017).

People who are extraverted are assumed to exhibit sociability, affection, spontaneity, and be generally friendly (McCrae and Costa, 1987). They may also be described as outgoing and energetic (Bastian et al., 2017). According to SEL, effective communication, standing up for others and leadership are key relationship skills which are likely exhibited by extroverts (CASEL, 2020). Importantly, extraversion has shown some significant positive correlations with satisfaction and job affect in several organizational studies (Judge et al., 2002; Zimmerman, 2008; Avery et al., 2015), which may be explained in part by the effort made by extraverts to interact with colleagues, similar to agreeableness. There is also some evidence that extraversion correlates negatively with intentions to quit (Sarwar et al., 2013), but also evidence of a small *positive* correlation with turnover (Timmerman, 2006). Whether extraversion correlates with satisfaction in teachers is unclear, however, it did not predict retention in first year United States teachers (Bastian et al., 2017).

Maintaining socio-emotional rapport with students and colleagues may also be enhanced by possessing the trait of honesty-humility, because people who are honest and humble are assumed to be sincere, fair, modest, and avoid greed (Ashton and Lee, 2009). According to SEL, these characteristics are important for recognizing one’s strengths and limitations (e.g., self-awareness), which also facilitate a grounded sense of confidence and purpose (CASEL, 2020). Honesty-humility has also been positioned as a sixth personality trait with considerable evidence showing that it better predicts job performance (positively) and workplace delinquency (negatively) than the original Big Five factors (Ashton and Lee, 2005; Zettler and Hilbig, 2010; Johnson et al., 2011; Ashton et al., 2014; Wiltshire et al., 2014; de Vries and van Gelder, 2015). Importantly, the honesty-humility personality dimension positively correlates with both satisfaction and tenure (Wiltshire et al., 2014; Ceschi et al., 2016). Given the clear value of honesty-humility as a predictor of workplace outcomes in organizational research, and the absence of research measuring this trait in teachers, it was included in the present study.

In contrast to these socially adaptive attributes, there are also socially maladaptive attributes which could undermine wellbeing and sustainability in the workplace. For instance, people who are *neurotic* are assumed to exhibit worry, nervousness, insecurity, and be emotionally unstable (McCrae and Costa, 1987). They may also be described as impatient and vulnerable (Bastian et al., 2017), which likely undermines their self-efficacy and increases their risk of burnout or turnover (Maslach et al., 2001). While neuroticism has shown some significant negative correlations with satisfaction in several studies (Judge et al., 2001, 2002; Matzler and Renzl, 2007; Zimmerman, 2008), there is also evidence of no association (Furnham et al., 2002; Furnham, 2009; Avery et al., 2015). The negative associations may be explained, in part, by the propensity of neurotic individuals to negatively appraise situations and events, although the mixed findings suggest that this effect could be job and or/industry dependent. There is also some evidence of modest positive correlations between neuroticism and intentions to quit (Zimmerman, 2008; Sarwar et al., 2013), as well as turnover (Salgado, 2002; Zimmerman, 2008; Swider and Zimmerman, 2010; Rubenstein et al., 2018), but not tenure (Burke and Witt, 2004). Whether neuroticism correlates with satisfaction in teachers is unclear, however, it did not predict retention in first year United States teachers (Bastian et al., 2017).

According to the SEL framework, the competency of self-management involves setting goals, planning, organization and taking initiative (CASEL, 2020). Self-management may be particularly important in international schools because resources and support may be limited, requiring teachers to be more self-sufficient and proactive. As such, teachers high in *conscientiousness* and *innovation* may be better positioned to function optimally in these environments. This is partly because highly conscientious teachers are also assumed to possess greater self-efficacy (Martocchio and Judge, 1997; van Daal et al., 2014), which is the conviction that they can successfully attain their goals (Bandura, 1977). People who are conscientious are assumed to exhibit self-discipline, perseverance, reliability and are generally well-organized (McCrae and Costa, 1987). They may also be described as goal-oriented and hardworking (Mount et al., 2006; Swider and Zimmerman, 2010). In accordance with self-efficacy theory, it is these self-regulating behaviors that enables those high in conscientiousness to be productive (Bandura, 1977). Teacher self-efficacy is also argued to be particularly important for protecting against stress and burnout because of an enhanced sense of competency and effective coping (Schwarzer and Hallum, 2008). Conscientiousness typically outperforms other Big Five personality traits as a predictor of workplace outcomes, presumably because of its direct role in enhancing performance and subsequent achievement (Organ and Lingl, 1995; Barrick et al., 2002; Zimmerman, 2008). Conscientiousness has shown consistent significant positive correlations with satisfaction in several organizational studies (Organ and Lingl, 1995; Furnham et al., 2002; Judge et al., 2002; Furnham, 2009; Ilies et al., 2009), which may be explained in part by the effort made by conscientious individuals to earn formal (e.g., remuneration) and informal (e.g., respect) work-related reward (Judge et al., 2002). Similarly, there is some evidence for a consistent, albeit weaker, negative association between conscientiousness and turnover and turnover intentions (Barrick et al., 2002; Salgado, 2002; Zimmerman, 2008; Swider and Zimmerman, 2010; Sarwar et al., 2013; Agarwal and Gupta, 2018; Rubenstein et al., 2018). Importantly, conscientiousness predicted higher retention rates in first year United States teachers (Bastian et al., 2017).

Innovation has been described as an important feature of creativity because it involves developing new methods of practice (Pulakos et al., 2000). It has also been defined as a form of proactive personality, which involves taking initiative, improving current circumstances and challenging the *status quo* rather than being passive (Crant, 2000). Studies examining proactive personality have shown that it correlates positively with satisfaction (Seibert, 1999; Fuller and Marler, 2009; Li et al., 2010, 2017) but only weakly negatively correlates with turnover intentions (Joo et al., 2015). It has yet to be determined if these associations would also be found in teachers.

Researchers agree job stressors deplete teacher energy which eventually diminishes their coping resources (Blase, 1982; Jennings et al., 2017). While more experienced teachers are assumed to have developed a greater variety of coping resources, these resources are still vulnerable to erosion, which may eventually lead to burnout. That is, a growing discrepancy between coping resources and work demands results in stress (Blase, 1982; Maslach et al., 2001). There is consistent evidence that teachers are vulnerable to experiencing high levels of stress and burnout (Kyriacou, 2001; Chaplain, 2008; Schwarzer and Hallum, 2008; Yu et al., 2015). As such, dispositional attributes which preserve coping resources are fundamental for sustaining teachers in meeting necessary workplace demands. This is particularly true for teachers in international schools who may experience less stability amongst their students and colleagues whilst also being at greater risk of feeling isolated (Hayden and Thompson, 2008; Bunnell, 2019). Within the SEL framework, the self-management competency points toward emotional regulation and the use of stress management strategies (CASEL, 2020). Accordingly, *resilience* and *stress tolerance* are likely to be correlates of both satisfaction and position duration in teachers (Gu and Day, 2007). Resilience can be described as the capacity to rebound (bounce back) from adversity (Luthans, 2002) and has been argued to represent one of the most important positive resources (or “psychological capital”) when placed in a stressful workplace (Avey et al., 2009). Those exhibiting resilience are assumed to engage in realistic assessments and quickly develop coping strategies to manage the challenges they face (Coutu, 2002). Resilience has been shown to consistently positively correlate with job satisfaction (Youssef and Luthans, 2007; Hudgins, 2016; Zheng et al., 2017) and negatively correlate with quitting intentions (Avey et al., 2009; Hudgins, 2016). While there has been considerable research interest in teacher resilience (e.g., Howard and Johnson, 2004; Beltman et al., 2011; Hong, 2012; Mansfield et al., 2012; Gu and Day, 2013), there is a paucity of studies examining the role of teacher resilience on satisfaction and retention, despite resilience being an apparently natural influence on an appointment’s duration. As such, we chose to include resilience in the current study. We also chose to include stress tolerance because stress has been found to negatively correlate with satisfaction (Kyriacou and Sutcliffe, 1979; Pearson and Moomaw, 2005; Conley and You, 2009; Klassen et al., 2010; Sass Daniel, 2011; Collie, 2012), and positively correlate with intentions to quit (Sass Daniel, 2011; Ryan et al., 2017).

Self-determination theory is one of the leading psychological theories of general motivation, whereby autonomous (*intrinsic*) and controlled (*extrinsic*) motivation are used to explain goal-orientation (Deci and Ryan, 1985). Intrinsic motivation can be described as self-determined and autonomous, typically characterized by the pursuit of goals that are inherently interesting or satisfying (Ryan and Deci, 2000). Extrinsic motivation, by contrast, can be described as non-self-determined and controlled, typically characterized by the pursuit of goals that offer external reward (e.g., financial) or to avoid punishment (Ryan and Deci, 2000). It can also be argued that these motivational inclinations fit within the SEL framework, specifically for self-management and self-awareness, whereby self-agency, setting personal goals, developing interests and a sense of purpose are considered valuable (CASEL, 2020). While there is a wide body of research examining the impact of teachers on the intrinsic and extrinsic motivation in school students (e.g., Noels et al., 1999; Patrick et al., 2000; Lam et al., 2008; Kusurkar et al., 2011), there is little data available on these motivational dimensions in teachers and their associations with satisfaction and retention. However, some evidence suggests a very strong positive association between intrinsic motivation and satisfaction in teachers (Shah et al., 2012; Raza et al., 2015). Several studies examining retention across other industries have found that internal locus of control (similar to intrinsic motivation) correlates positively with satisfaction (Judge et al., 2001; Srivastava, 2013) and negatively with intentions to quit (Spector and Michaels, 1986; Rubenstein et al., 2018). As such, there is some evidence to expect an association between intrinsic motivation and satisfaction and position duration. By contrast, extrinsic motivation involves a continual focus on extrinsic pressure and reinforcement, which can diminish the feelings of joy, enthusiasm and interest in the task, thus reducing the likelihood of experiencing satisfaction in educational settings (Niemiec and Ryan, 2009). People who are sensitive to reinforcement may also exhibit *impulsivity* which may lead to spontaneous or affect-driven, quitting without fully evaluating the longer-term consequences (e.g., seeking higher remuneration but lower job security) (Holtom et al., 2008; Zimmerman, 2008; Hom et al., 2012; Krupić and Corr, 2020). Indeed, research has shown that there is a positive association between turnover intentions and turnover only in those with low risk aversion, suggesting that impulsivity moderates the risk of following through on quitting intentions (Allen et al., 2005). Research has yet to examine the role of impulsivity when predicting satisfaction and position duration in teachers, which could be useful when developing risk profiles for job candidates. Thus it is plausible to assume, theoretically, that extrinsic motivation might be a negative correlate of workplace satisfaction and impulsivity might be a negative correlate of position duration.

Finally, and in light of the particularly demanding and potentially unstable circumstances faced by teachers in international schools, we included two further attitudinal measures which could be self-protecting in these contexts. Given that teachers in international schools are at greater risk of isolation and lower support, they may adopt certain organizational attitudes to enhance their workplace security and wellbeing. Specifically, *subordination* and *organizational commitment* might be relevant because of their role in maintaining job security particularly in settings where teachers are heavily reliant on school management for support, training and, inevitably, a favorable recommendation. Subordination, or submissiveness to authority, while infrequently studied (e.g., Petersen and Dietz, 2008; Brannan, 2015), is a trait which could be adaptive in some organizational settings (DeZoort and Roskos-Ewoldsen, 1997). Specifically, yielding to authority is one strategy for maintaining rapport with management, which may offer some protection during periods of instability. Likewise, adopting a sense of obligation to an organization, or normative commitment, is another strategy for enhancing job security. Organizational commitment is one of the most commonly measured variables in organizational research and has close links to both satisfaction and retention. Importantly, in teachers, organizational commitment correlates positively with satisfaction and negatively with intentions to quit (Conley and You, 2009; Zeinabadi, 2010; Bos et al., 2016).

In summary, we have explored educational, motivational and organizational frameworks as a means to understand (and potentially predict) key markers of placement success in teachers. We have identified 15 possible correlates of workplace satisfaction and position duration which may be important for understanding how and why teachers might experience satisfaction within their workplace and remain in their position. Specifically, empathy (cognitive and emotional), agreeableness, extraversion, honesty-humility, neuroticism, conscientiousness, innovation, resilience, stress tolerance, intrinsic motivation, extrinsic motivation, impulsivity, subordination, and (normative) organizational commitment were measured in the present study.

### The Present Study

The purpose of the present study was to identify and measure psychological attributes that correlate with two markers of placement success in teachers; workplace satisfaction and position duration. This was achieved by first reviewing the literature for relevant traits and then measuring them in a cross-sectional design using validated instruments in a sample of international schoolteachers. We also acknowledged that school levels (e.g., high school and elementary) are often researched individually (e.g., Montgomery and Rupp, 2005; Näring et al., 2012; van Daal et al., 2014; Li et al., 2017), with some evidence that different patterns of findings emerge when analyzed separately (e.g., Kukla-Acevedo, 2009; Hughes, 2012). As such, we repeated our analyses to explore the hypothesized associations at each school level if they were adequately represented in our sample. Importantly, our sample consisted of teachers placed within international schools, who are not only under-researched but are also especially challenging from a recruitment perspective because they are at high risk of short appointments due to the additional complexity of their placement demands (e.g., unfamiliar country/culture and relocation requirements etc.) (Odland and Ruzicka, 2009; Chandler, 2010; Mancuso et al., 2010; Bunnell, 2019). As such, identifying valuable correlates in this group of teachers is anticipated to be particularly useful to those involved in international placement decisions.

Following a review of the literature, it was clear that there were several possible correlates with satisfaction and position duration in teachers. In developing our hypotheses, we chose to identify the most likely correlates in the present study, particularly where there was theoretical or empirical evidence that they are relevant to teachers. Following correlational analysis, we also subjected these variables to regression and neural network analyses to determine if these variables offered any potential predictive utility. The neural network models were included to, in part, further support any linear associations observed in the regression analyses but also explore any potential non-linearity that may exist amongst these variables. While these non-linear analyses were exploratory, we have provided hypotheses for linear analyses as per the theoretical and empirical framework:

H1. For workplace satisfaction, it was expected that (a) agreeableness, (b) conscientiousness, (c) extraversion, (d) honesty-humility, (e) empathy, (f) intrinsic motivation, (g) innovation, (h) work stress tolerance, (i) organizational commitment, and (j) resilience would be positive correlates, after controlling for age, gender and socially desirable responding.

H2. For workplace satisfaction, it was also expected that (a) neuroticism and (b) extrinsic motivation would be negative correlates, after controlling for age, gender and socially desirable responding.

H3. For position duration, it was expected that (a) conscientiousness, (b) honesty-humility, (c) intrinsic motivation, (d) work stress tolerance, (e) organizational commitment, and (f) resilience would be positive correlates, after controlling for age, gender and socially desirable responding.

H4. For position duration, it was also expected that (a) neuroticism and (b) impulsivity would be negative correlates, after controlling for age, gender and socially desirable responding.

## Materials and Methods

### Participants

**FIGURE 1:**
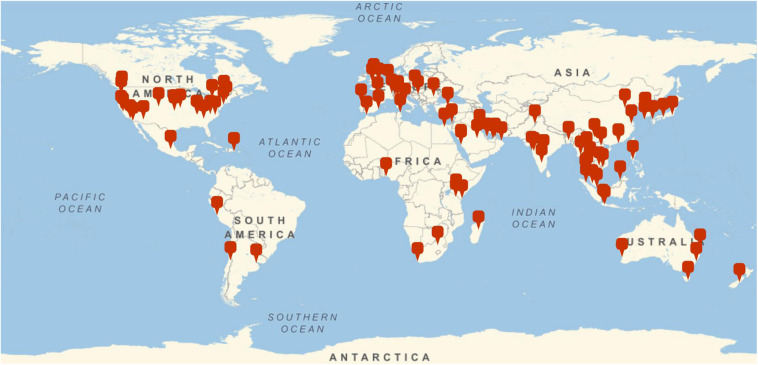
Geographical distribution of the sample.

**FIGURE 2:**
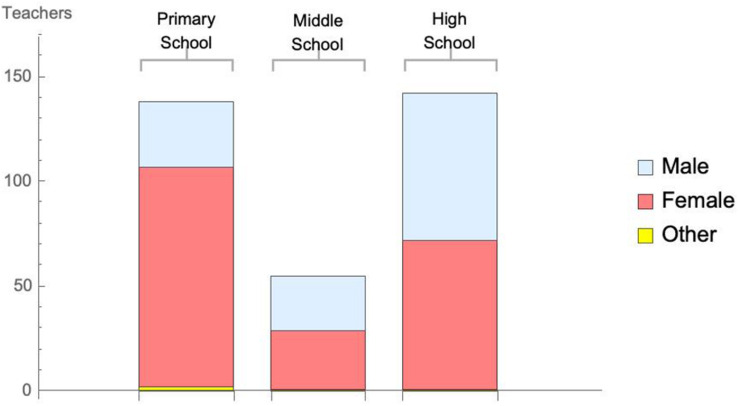
Distribution of sample according to gender and school level.

Participants were 335 teachers (61% female), aged between 18 and 74 years (*M* = 42.21, *SD* = 9.08), employed across various international locations. Most of the sample were currently working in Asia (46%), with 35.5% in the middle east, 4.5% in Europe, 4.2% in North America, 3.3% in in South America, 1.5% in Oceania and less than 2% in central America and the Caribbean combined. The location of teaching placements is illustrated in Figure 1, while the breakdown of school level to gender is illustrated in Figure 2. The majority of participants held a Bachelor degree (59.7%), with 55.2% holding a Masters qualification, 24.2% holding a diploma, and 3% holding a Doctorate.

### Measures

#### Big Five Personality Dimensions

Four (extraversion, agreeableness conscientiousness, and neuroticism) of the Big Five personality dimensions were measured using the Mini-International Personality Item Pool (IPIP; Donnellan et al., 2006). This scale uses four items per factor with items rated on a five-point Likert scale (*strongly disagree* to *strongly agree*). The mini-IPIP shows comparable, and in some cases better, psychometric properties when compared to Big Five inventories with more items (Crede et al., 2012). In the current study, internal consistency estimates were acceptable for extraversion (0.77) and agreeableness (0.70), but lower than ideal for conscientiousness (0.65), and neuroticism (0.64).

#### Subordination

Subordination was measured using the Submissiveness to Organizational Authority Scale developed by DeZoort and Roskos-Ewoldsen (1997). The 10 items reflected the extent to which an employee follows directions from authority. The items were rated on a seven-point Likert scale anchored with *strongly disagree* to *strongly agree.* In the current study, this scale yielded acceptable internal consistency (0.73).

#### Organizational Commitment

Organizational commitment was measured using six items from the Normative Commitment Scale developed by Allen and Meyer (1990). The items reflected the extent to which an employee endorses moving between organizations and their attitudes about organizational loyalty. The items were rated on a seven-point Likert scale anchored with *strongly disagree* to *strongly agree.* In the current study, this scale yielded marginally acceptable internal consistency (0.69).

#### Empathy

Empathy was measured using two subscales from the Brief Form of the Interpersonal Reactivity Index (B-IRI; Ingoglia et al., 2016). One subscale, perspective taking, represented a form of cognitive empathy whereby a person has the capacity to adopt another’s viewpoint. The other subscale, empathic concern, represented a form of emotional empathy whereby a person feels sympathy and concern for others. Each subscale was measured using four items on a 5-point scale anchored by *does not describe me well* to *describes me very well*. Scores were calculated separately for each subscale by averaging the item ratings, with higher scores indicating greater cognitive and emotional empathy. In the current study, both the perspective taking (0.84) and empathic concern (0.79) subscales yielded acceptable internal consistency estimates.

#### Honesty-Humility

Honesty and humility were measured using the honesty-humility subscale from the HEXACO-PI-R developed by Ashton and Lee (2009). Ashton and Lee (2009) argue that this subscale captures a trait that is only peripherally represented in Big Five inventories. The 10 items reflected a person’s sincerity, fairness, modesty and greed avoidance. The items were rated on a five-point Likert scale anchored with *strongly disagree* to *strongly agree.* In the current study, this subscale yielded a lower than ideal internal consistency (0.63).

#### Intrinsic and Extrinsic Motivation

Intrinsic and extrinsic motivation, sometimes termed autonomous and controlled motivation, respectively, were measured using two subscales from the Work Extrinsic and Intrinsic Motivation Scale developed by Tremblay et al. (2009). For the purpose of brevity, we only used the three-item intrinsic and the three-item extrinsic subscales of this instrument. We also varied the preceding instructions for responding to the items to specifically refer to why they are involved in “teaching” rather than just “work.” The intrinsic items were designed to capture the inherent satisfaction or pleasure associated with teaching, whereas the extrinsic items were designed to capture the relevance of external reinforcers (e.g., income, security). The items were rated on a seven-point scale anchored with *does not correspond to me at all* to *corresponds exactly.* In the current study, both the extrinsic (0.77) and intrinsic (0.83) subscales yielded acceptable internal consistency estimates.

#### Innovation and Stress Tolerance

Innovation and stress tolerance were measured with two subscales taken from the Workplace Adaptive Performance Scale developed by Charbonnier-Voirin and Roussel (2012). Innovation, measured with four items (from the creatively subscale), reflected the extent to which an individual is willing to propose and develop new methods of working. Stress tolerance, measured with three items, reflected the ability to remain calm under conditions of duress at work. The items were rated on a seven-point Likert scale anchored with *strongly disagree* to *strongly agree.* In the current study, both the innovation (0.77) and stress management (0.83) subscales yielded acceptable internal consistency estimates.

#### Resilience

Resilience was measured using the 15-item Protective Factors for Resilience Scale developed by Harms et al. (2017). These items reflected the extent to which a person has the resources (personal and social) to respond to challenges. The items were rated on a seven-point scale anchored with *strongly disagree* to *strongly agree.* In the current study, this measure yielded good internal consistency (0.79). As the resilience items were only added partway through the data collection, only 236 participants completed this scale.

#### Impulsivity

Impulsivity was measured with the Brief Barratt Impulsivity Scale developed by Steinberg et al. (2013), based upon items from the 11th version of the BIS (Patton et al., 1995). The eight items reflected a person’s tendency to act quickly and without forethought. The items were rated on a four-point scale anchored with *rarely/never* to *almost always.* In the current study, this subscale yielded good internal consistency (0.79).

#### Workplace Satisfaction and Position Duration

Workplace satisfaction was measured using a single item; How satisfied are you in your current work place? rated on a seven-point sale anchored with *extremely unsatisfied* to *extremely satisfied*. Position duration was measured by asking participants to indicate how long they had been in their current position in years and months. This was converted into a single continuous measure of years for analysis.

#### Socially Desirable Responding

Because of the heavy reliance on self-report in the present study, and because this methodology might be used in recruitment contexts (where responses may not be anonymous and faking good responses might be elevated), we controlled for socially desirable responding in our analyses using the short form of the Balanced Inventory of Desirable Responding developed by Hart et al. (2015). The 16 items were selected from the longer form developed by Paulhus (1984, 1991) and captured overly positive responding (self-deceptive enhancement) and biases toward pleasing others (impression management). We opted to exclude one item (I have sometimes doubted my ability as a lover) because of its irrelevance to the workplace, and the potential to make participants feel unnecessarily uncomfortable. This left 15 items rated on a seven-point scale anchored with *not true* to *very true*. In the current study, while the self-deceptive enhancement scale showed low internal consistency (0.58), the impression management scale yielded acceptable internal consistency (0.70). Low estimates of internal consistency for self-deceptive enhancement are a known criticism of this construct (Hart et al., 2015).

### Procedure

An online survey battery including all the measures was distributed via the client networks and social media page of an Australian teacher recruitment company, which assists in approximately 2,000 international teacher placements per annum. The link to the survey was emailed by employees in the recruitment company to known international school leaders, who were encouraged to distribute it to their current teaching staff at their discretion. The survey link was essentially snowballed throughout the networks of approximately 8–10 international schools and on social media. For participants who completed the survey via social media, they were offered the chance to enter a prize draw for a gift card. The instruments in the survey were randomly counterbalanced to mitigate any order or fatigue effects. Participant consent was secured using a check box (following a full information page) which would prevent continuation of the survey unless consent was indicated. All data were collected anonymously. The survey took approximately 30–40 min to complete. These procedures were approved by the University Human Research Ethics Committee.

### Data Analysis

Independent *t*-tests were used to compare teacher scores between gender and school levels. To test the hypotheses, hierarchical regression analyses were performed separately with workplace satisfaction and position duration as dependent variables. Age, gender and socially desirable responding measures were controlled for at Step 1 and all the psychological attributes were entered at Step 2. Regression assumptions of normally distributed residuals, absence of collinearity and an absence of influential cases were satisfied. However, there was evidence of heteroscedasticity for the position duration regression analyses and for workplace satisfaction in high school teachers. In these instances, wild bootstrapping (2,000 resamples) was utilized to estimate unbiased confidence intervals. To simplify the interpretation of the gender variable, all gender associations excluded participants who did not identify as male or female (*N* = 4).

Given the exploratory nature of this investigation, we share the vision of a “world beyond *p* < 0.05” (Wasserstein et al., 2019) and adopt the sentiments of Cumming (2013) to, instead of interpreting traditional *p*-values, focus primarily on effect sizes (*r*, β, and *d*) and confidence intervals when interpreting the results. We believe this is particularly important in large samples where trivial effects can be over-interpreted when arbitrary and dichotomous criteria are applied. Specifically, and according to meta-analytically derived recommendations by Gignac and Szodorai (2016) for individual differences researchers, *r* and β values of 0.10, 0.20, and 0.30 and *d-*values of 0.20, 0.40, and 0.60 were considered relatively small, typical and relatively large, respectively^1^. In this investigation, we choose to only interpret typical to large effects as meaningful.

We also recognize that there are further means for assessing whether a variable is “meaningful”. As such, and based on observed correlations and hierarchical regression outcomes, a subset of psychometric (non-demographic) variables were selected as potential correlates of workplace satisfaction and position duration in four neural network models (at both primary/elementary and high school levels). Neural Networks (NN) is a branch of machine learning, now more accessible due to algorithmic and technological advances in GPU speeds. Such approaches are being deployed across fields such as medicine, finance, business, robotics, physics and indeed any field with large data volumes. While applications in psychology are less well-developed, interest is growing given NN’s potential to address the field’s long-standing reproducibility issues along with added flexibility in the types of models it is able to accommodate (Orrù et al., 2020).

By adopting a neural network modeling approach, directions from the theoretical framework, effect sizes and confidence intervals can be translated into tentative predictions that can both further inform theory as well as offering direct applicability for school’s teacher-placements. The intent was to, with suitable caveats regarding the cross-sectional nature of the data and relatively modest sample sizes, detect suggestive cause-effect (non-linear) relationships between psychological attributes and the dependent variables of position duration and workplace satisfaction. These neural network models offer a more detailed understanding of how (normalized) individual psychometric attributes can influence (normalized) measures of workplace satisfaction and position duration. In particular, departures from linearity are detectable when graduated effects exist depending on the degree of a psychological attribute possessed by a teacher (from 0 to 3 standard deviation units). These models are also valuable for supporting any linear assumptions found in the regression analyses.

## Results

### Gender and School Level Comparisons

Independent samples *t*-tests were used to compare gender on each of the attributes and placement markers. Females scored higher than males on empathic concern, *t*(329) = −3.08, *d* = −0.34, and honesty-humility, *t*(329) = −2.80, *d* = −0.31, but the effect sizes were all small to typical. One-way ANOVAs were used to compare teachers at different school levels (primary, middle and high) on each of the attributes and the job fit markers. None of these comparisons were interpreted to be meaningfully different, thus teachers were regarded as homogenous in their psychological profiles and markers of placement success across school levels. The descriptive statistics, reported separately by gender and school level, for all of the measured attributes are presented in Table 1.

**TABLE 1:** Descriptive statistics for all variables, separated by gender and school level.

	All *M* (*SD*)	Gender *M* (*SD*)		School level *M* (*SD*)		
Attribute	All (*N* = 335)	Men (*N* = 127)	Women (*N* = 204)	Primary (*N* = 138)	Middle (*N* = 56)	High (*N* = 141)
Agreeableness	4.04 (0.69)	3.94 (0.74)	4.11 (0.65)	4.04 (0.66)	4.01 (0.77)	4.05 (0.70)
Conscientiousness	3.77 (0.76)	3.80 (0.73)	3.75 (0.77)	3.79 (0.69)	3.72 (0.82)	3.76 (0.80)
Extraversion	3.03 (0.90)	3.08 (0.88)	3.00 (0.92)	3.06 (0.85)	2.95 (0.97)	3.02 (0.93)
Neuroticism	2.48 (0.79)	2.42 (0.78)	2.51 (0.79)	2.53 (0.77)	2.56 (0.85)	2.39 (0.78)
Empathy – EC	4.15 (0.69)	4.01 (0.70)	4.25 (0.67)	4.16 (0.69)	4.21 (0.71)	4.12 (0.70)
Empathy – PT	3.94 (0.73)	3.90 (0.73)	3.98 (0.73)	3.91 (0.80)	3.94 (0.70)	3.97 (0.68)
Innovation	5.72 (0.78)	5.77 (0.81)	5.71 (0.71)	5.78 (0.68)	5.46 (0.88)	5.76 (0.81)
Stress tolerance	5.71 (0.92)	5.81 (0.85)	5.67 (0.93)	5.74 (0.88)	5.51 (1.06)	5.74 (0.91)
Intrinsic motivation	5.77 (1.02)	5.78 (0.96)	5.79 (1.03)	5.84 (0.99)	5.55 (1.02)	5.77 (1.05)
Extrinsic motivation	4.45 (1.37)	4.49 (1.43)	4.45 (1.34)	4.36 (1.34)	4.48 (1.33)	4.52 (1.43)
Subordination	4.09 (0.78)	4.06 (0.77)	4.12 (0.78)	4.14 (0.71)	4.18 (0.85)	4.01 (0.80)
Organizational commitment	3.67 (1.00)	3.84 (1.04)	3.55 (0.97)	3.64 (0.96)	3.63 (0.96)	3.71 (1.06)
Honesty-humility	3.97 (0.57)	3.86 (0.59)	4.04 (0.55)	4.00 (0.57)	3.80 (0.50)	3.99 (0.60)
Resilience^†^	4.57 (0.53)	4.49 (0.52)	4.61 (0.53)	4.59 (0.54)	4.38 (0.62)	4.61 (0.47)
Workplace satisfaction	4.74 (1.62)	4.80 (1.57)	4.72 (1.66)	4.66 (1.73)	4.80 (1.53)	4.80 (1.55)
Position duration (years)	5.46 (5.13)	5.56 (5.02)	5.40 (5.27)	6.00 (5.77)	4.22 (3.49)	5.40 (4.94)

*Four participants did not identify as male or female were thus excluded from the separate gender descriptive statistics. ^†^*n* = 236.*

### Correlational Analyses

Pearson correlations between all the variables have been reported in Table 2. The largest positive associations occurred between stress tolerance and innovation (*r* = 0.58), empathy and agreeableness (*r*_*EC*_ = 0.54; *r*_*PT*_ = 0.48), and between intrinsic motivation and innovation (*r* = 0.43). The largest negative associations occurred between impulsivity and conscientiousness (*r* = −0.48), between stress tolerance and impulsivity (*r* = 0.46) and between resilience and neuroticism (*r* = −0.47). Workplace satisfaction showed only one typical-sized negative association (*r* = −0.21) with neuroticism. By contrast, position duration showed a typical to large positive association with commitment (*r* = 0.25). Note that workplace satisfaction and position duration were not correlated (*r* = 0.07).

**TABLE 2:** Pearson Correlations between All Variables.

Variable	1	2	3	4	5	6	7	8	9	10	11	12	13	14	15	16	17	18
1. Gender	−																	
2. Age	0.00	−																
3. Agreeableness	0.12	0.10	−															
4. Conscientiousness	-0.03	0.15	0.17	−														
5. Extraversion	-0.04	-0.03	**0**.**26**	-0.09	−													
6. Neuroticism	0.06	-0.10	-0.12	-0.17	0.04	−												
7. Impulsivity	0.05	-0.06	**−0**.**21**	**−0**.**48**	0.17	**0**.**34**	−											
8. Empathy – EC	0.17	0.10	**0**.**54**	0.07	0.12	-0.05	-0.16	−										
9. Empathy – PT	0.05	0.10	**0**.**48**	**0**.**20**	0.03	**−0**.**26**	**0**.**38**	**0**.**59**	−									
10. Innovation	-0.04	0.05	0.17	0.18	0.12	-0.13	**−0**.**37**	**0**.**21**	**0**.**23**	−								
11. Stress Tolerance	-0.08	0.07	**0**.**28**	**0**.**32**	0.04	**−0**.**41**	**−0**.**46**	**0**.**25**	**0**.**46**	**0**.**58**	−							
12. Intrinsic	0.01	0.05	**0**.**27**	0.13	0.15	-0.04	0.14	**0**.**24**	**0**.**24**	**0**.**43**	**0**.**30**	−						
13. Extrinsic	-0.01	0.08	-0.10	0.03	0.01	0.00	0.06	-0.03	-0.04	0.02	0.03	0.17	−					
14. Subordination	0.04	0.05	0.17	**0**.**22**	-0.04	-0.14	0.19	0.05	0.17	-0.10	0.10	0.10	0.01	−				
15. Organizational Commitment	-0.14	-0.05	0.05	0.19	0.04	-0.01	-0.16	0.05	0.18	-0.01	0.14	0.12	0.00	**0**.**26**	−			
16. Honesty-humility	0.15	0.14	**0**.**23**	**0**.**20**	-0.15	**−0**.**24**	**−0**.**33**	**0**.**23**	**0**.**24**	0.15	0.15	0.09	**−0**.**21**	0.08	-0.05	−		
17. Resilience^†^	0.11	-0.02	**0**.**28**	0.03	**0**.**25**	**−0**.**47**	**−0**.**22**	0.10	**0**.**25**	**0**.**30**	**0**.**23**	**0**.**38**	0.08	0.01	-0.03	0.14	−	
18. Workplace Satisfaction	-0.02	0.06	-0.02	0.04	0.02	**−0**.**21**	-0.10	-0.04	0.08	-0.01	0.11	0.09	0.05	0.13	0.13	0.08	0.14	−
19. Position Duration (years)	-0.01	0.18	-0.09	0.04	0.03	0.02	-0.10	0.06	0.11	0.08	0.10	0.11	0.06	0.07	**0**.**23**	-0.12	0.01	0.07

*N = 335, ^†^*n* = 236, boldface indicates typical to large effect sizes; EC, empathic concern; PT, perspective taking.*

To test the hypotheses and explore the correlational strength of the psychological attributes, hierarchical regression analyses were performed separately with workplace satisfaction (Table 3) and position duration (Table 4) as dependent variables. Age, gender and socially desirable responding measures were controlled for at Step 1 and all the psychological attributes were entered at Step 2. As resilience was only added partway through the data collection, it was not included in the reported regression analyses. However, separate regressions revealed that it did not show meaningful associations with workplace satisfaction or position duration for the subsample.

**TABLE 3:** Hierarchical regression predicting workplace satisfaction across all school levels.

		B [95% CI]	β	Adj. R^2^	Δ R^2^
**Step 1**				0.01	0.02
	Age	0.01 [−0.01, 0.03]	0.05		
	Gender	-0.09 [−0.45, 0.27]	-0.03		
	Self-deceptive enhancement	0.06 [−0.18, 0.29]	0.03		
	Impression management	0.20 [0.01, 0.39]	0.12		
**Step 2**				0.05	0.08
	Agreeableness	-0.27 [−0.60 0.06]	-0.11		
	Conscientiousness	-0.10 [−0.37 0.18]	-0.04		
	Extraversion	0.14 [−0.08, 0.35]	0.08		
	Neuroticism	-0.39 [−0.65, -0.13]	-0.19		
	Impulsivity	-0.13 [−0.68, 0.42]	-0.04		
	Empathy – EC	-0.21 [−0.55, 0.14]	-0.09		
	Empathy – PT	-0.13 [−0.21, 0.47]	0.06		
	Innovation	-0.22 [−0.54, 0.09]	-0.10		
	Stress tolerance	0.06 [−0.22, 0.34]	0.03		
	Intrinsic motivation	0.17 [−0.04, 0.37]	0.10		
	Extrinsic motivation	0.04 [−0.10, 0.17]	0.03		
	Subordination	0.12 [−0.13, 0.37]	0.06		
	Organizational commitment	0.18 [−0.01, 0.37]	0.11		
	Honesty-humility	0.14 [−0.22, 0.51]	0.05		

*N = 331, boldface indicates typical to large effect sizes; EC, empathic concern; PT, perspective taking.*

**TABLE 4:** Hierarchical regression predicting position duration across all school levels with confidence intervals estimated via wild bootstrapping.

		B [95% CI]	β	Adj. R^2^	Δ R^2^
**Step 1**				0.05	0.06
	Age	0.11 [0.05, 0.17]	0.19		
	Gender	0.26 [−0.86, 1.39]	0.03		
	Self-deceptive enhancement	1.01 [0.29, 1.74]	0.16		
	Impression management	0.00 [−0.59, 0.60]	0.00		
**Step 2**				0.14	0.13
	Agreeableness	-1.80 [−2.79, -0.80]	**−0**.**24**		
	Conscientiousness	-0.47 [−1.30, 0.37]	-0.07		
	Extraversion	0.27 [−0.37, 0.91]	0.05		
	Neuroticism	0.52 [−0.27, 1.31]	0.08		
	Impulsivity	-1.31 [−2.96, 0.35]	-0.11		
	Empathy – EC	0.77 [−0.27, 1.80]	0.10		
	Empathy – PT	0.42 [−0.62, 1.45]	0.06		
	Innovation	0.06 [−0.89, 1.01]	0.01		
	Stress tolerance	0.30 [−0.55, 1.14]	0.05		
	Intrinsic motivation	0.35 [−0.26, 0.96]	0.07		
	Extrinsic motivation	-0.07 [−0.47, 0.33]	-0.02		
	Subordination	0.16 [−0.57, 0.90]	0.02		
	Organizational commitment	0.91 [0.33, 1.48]	0.18		
	Honesty-humility	-1.65 [−2.75, -0.54]	-0.18		

*N = 331, boldface indicates typical to large effect sizes; EC, empathic concern; PT, perspective taking.*

As can be seen in Table 3, there were no variables which approximated typical to large effect sizes when using workplace satisfaction as the dependent variable. These results meant that none of the H1 or H2 expectations were supported with respect to the entire sample. As can be seen in Table 4. agreeableness was the only variable which approximated a typical or large effect size (β = −0.26) when using position duration as the dependent variable. These results meant that we found no support for H3 or H4 expectations. Next, to determine if there were any notable differences in the pattern of associations between school levels, we tested our hypotheses again by performing the same regression analyses separately for primary/elementary and high school teachers. Because the subsample of middle school teachers was too small for a regression with this many predictors, we excluded this group from these analyses. These subgroup regression analyses are reported in Tables 5–8.

**TABLE 5:** Hierarchical regression predicting workplace satisfaction in primary/elementary teachers.

		B [95% CI]	β	Adj. R^2^	Δ R^2^
Step 1				0.02	0.05
	Age	0.02 [−0.01, 0.05]	0.10		
	Gender	0.23 [−0.48, 0.93]	0.05		
	Self-deceptive enhancement	0.16 [−0.26, 0.57]	0.07		
	Impression management	0.26 [−0.06, 0.58]	0.15		
Step 2				0.05	0.13
	Agreeableness	-0.31 [−0.92, 0.29]	-0.12		
	Conscientiousness	-0.24 [−0.76, 0.29]	-0.09		
	Extraversion	0.23 [−0.16, 0.63]	0.11		
	Neuroticism	0.01 [−0.49, 0.51]	0.00		
	Impulsivity	0.06 [−1.03, 1.16]	-0.02		
	Empathy – EC	-0.53 [−1.15, 0.09]	**−0**.**21**		
	Empathy – PT	0.13 [−0.42, 0.68]	0.06		
	Innovation	-0.12 [−0.72, 0.48]	-0.05		
	Stress tolerance	0.36 [−0.14, 0.85]	0.18		
	Intrinsic motivation	0.09 [−0.28, 0.46]	0.05		
	Extrinsic motivation	-0.10 [−0.35, 0.14]	-0.08		
	Subordination	0.39 [−0.07, 0.84]	0.15		
	Organizational commitment	0.15 [−0.19, 0.49]	0.09		
	Honesty-humility	0.44 [−0.23, 1.10]	0.14		

*N = 138, boldface indicates typical to large effect sizes; EC, empathic concern; PT, perspective taking.*

**TABLE 6:** Hierarchical regression predicting position duration in primary/elementary teachers with confidence intervals estimated via wild bootstrapping.

		B [95% CI]	β	Adj. R^2^	Δ R^2^
Step 1				0.07	0.09
	Age	0.17 [−0.06, 0.28]	0.26		
	Gender	-0.47 [−2.82, 1.89]	-0.03		
	Self-deceptive enhancement	0.93 [−0.46, 2.32]	0.12		
	Impression management	-0.02 [−1.10, 1.05]	0.00		
Step 2				0.25	0.25
	Agreeableness	-3.53 [−5.37, -1.68]	**−0**.**39**		
	Conscientiousness	-1.50 [−3.11, -0.11]	-0.17		
	Extraversion	0.17 [−1.04, 1.38]	0.02		
	Neuroticism	0.74 [−0.78, 2.27]	0.10		
	Impulsivity	-3.99 [−7.33, -0.65]	**−0**.**28**		
	Empathy – EC	1.38 [0.52, 3.29]	0.16		
	Empathy – PT	0.72 [−0.98, 2.41]	0.10		
	Innovation	0.01 [−1.84, 1.82]	0.00		
	Stress tolerance	-0.08 [−1.59, 1.44]	-0.01		
	Intrinsic motivation	0.20 [−0.92, 1.32]	0.03		
	Extrinsic motivation	0.52 [−0.23, 1.27]	0.12		
	Subordination	1.21 [0.19, 2.61]	0.14		
	Organizational commitment	1.24 [−0.20, 2.27]	**0**.**20**		
	Honesty-humility	-1.32 [−3.35, 0.71]	-0.13		

*N = 138, boldface indicates typical to large effect sizes; EC, empathic concern; PT, perspective taking.*

**TABLE 7:** Hierarchical regression predicting workplace satisfaction in high school teachers with confidence intervals estimated via wild bootstrapping.

		B [95% CI]	β	Adj. R^2^	Δ R^2^
Step 1				0.00	0.03
	Age	0.00 [−0.03, 0.03]	-0.02		
	Gender	-0.31 [−0.83, 0.21]	-0.10		
	Self-deceptive enhancement	0.11 [−0.24, 0.46]	0.06		
	Impression management	0.13 [−0.16, 0.42]	0.09		
Step 2				0.05	0.14
	Agreeableness	-0.31 [−0.82, 0.20]	-0.14		
	Conscientiousness	0.12 [−0.30, 0.53]	0.06		
	Extraversion	0.13 [−0.20, 0.46]	0.08		
	Neuroticism	-0.65 [−1.03, -0.27]	**−0**.**33**		
	Impulsivity	-0.59 [−1.42, 0.25]	-0.17		
	Empathy – EC	0.15 [−0.36, 0.66]	0.07		
	Empathy – PT	0.10 [−0.47, 0.67]	0.04		
	Innovation	-0.29 [−0.76, 0.18]	-0.15		
	Stress tolerance	-0.13 [−0.56, 0.31]	-0.07		
	Intrinsic motivation	0.12 [−0.19, 0.43]	0.08		
	Extrinsic motivation	0.04 [−0.16, 0.24]	0.03		
	Subordination	-0.33 [−0.70, 0.04]	-0.17		
	Organizational commitment	-0.23 [−0.05, 0.51]	0.16		
	Honesty-humility	0.02 [−0.53, 0.57]	0.01		

*N = 141, boldface indicates typical to large effect sizes; EC, empathic concern, PT, perspective taking.*

**TABLE 8:** Hierarchical regression predicting position duration in high school teachers.

		B [95% CI]	β	Adj. R^2^	Δ R^2^
Step 1				0.04	0.06
	Age	0.07 [0.01, 0.20]	0.14		
	Gender	0.58 [−0.89, 2.68]	0.06		
	Self-deceptive enhancement	1.25 [0.14, 2.55]	0.22		
	Impression management	-0.16 [−1.10, 0.96]	-0.03		
Step 2				0.12	0.17
	Agreeableness	-0.21 [−1.93, 1.62]	-0.03		
	Conscientiousness	-0.37 [−1.09, 1.82]	-0.06		
	Extraversion	0.20 [−0.94, 1.95]	0.04		
	Neuroticism	0.96 [−0.48, 2.04]	0.16		
	Impulsivity	0.23 [−1.21, 5.25]	0.02		
	Empathy – EC	0.66 [−1.40, 2.07]	0.10		
	Empathy – PT	-1.54 [−3.80, 0.21]	**−0**.**22**		
	Innovation	0.65 [−3.15, 0.47]	-0.11		
	Stress tolerance	1.25 [0.33, 3.75]	**0**.**24**		
	Intrinsic motivation	0.49 [−0.55, 1.76]	0.11		
	Extrinsic motivation	-0.12 [−1.04, 0.35]	-0.04		
	Subordination	-0.72 [−2.04, 0.58]	-0.12		
	Organizational commitment	1.20 [0.57, 2.50]	**0**.**27**		
	Honesty-humility	-1.12 [−3.08, 1.09]	-0.14		

*N= 141, boldface indicates typical to large effect sizes; EC, empathic concern; PT, perspective taking.*

As can be seen in Table 5, empathic concern was the only variable which approximated a typical to large effect size (β = −0.21) when using workplace satisfaction as the dependent variable in primary/elementary school teachers, although this association was in the negative direction. As such, we found no support for the H1 or H2 hypotheses in this group. By contrast, and as can be seen in Table 6, three psychological factors (agreeableness, impulsivity, and organizational commitment) approximated typical to large effect sizes (β = −0.39, β = −0.28, β = 0.20, respectively) when using position duration as the dependent variable in primary/elementary school teachers. These results meant that H3 (e) was supported because organizational commitment was positively associated with position duration in primary/elementary school teachers. We also found support for H4 (b) because impulsivity was negatively associated with position duration in primary/elementary school teachers.

As can be seen in Table 7, neuroticism, was the only variable which approximated a typical to large effect size (β = −0.33) when using workplace satisfaction as the dependent variable in high school teachers. These results meant that we found support for H2 (a) in high school teachers. By contrast, and as can be seen in Table 8, perspective taking, stress tolerance and organizational commitment all approximated typical to large effect sizes (β = −0.22, β = 0.24, β = 0.27, respectively) when using position duration as the dependent variable in high school teachers. These results meant that we found support for H3 (d) and H3 (e) because stress tolerance and organizational commitment were both positively associated with position duration in high school teachers.

### Neural Network Models

**FIGURE 3:**
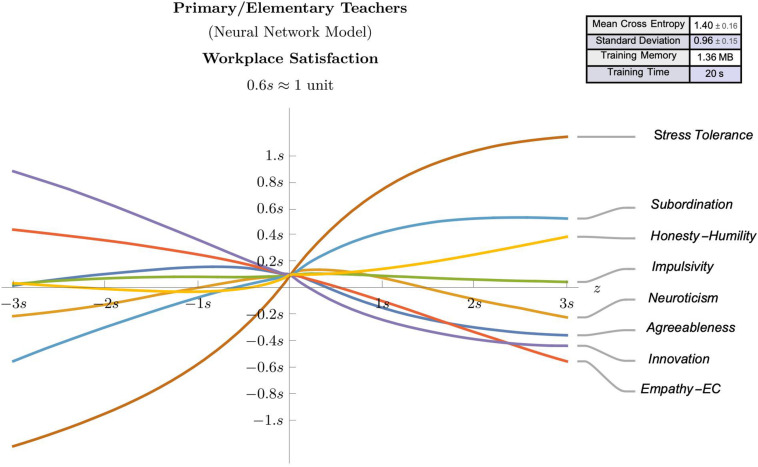
A (feed-forward) neural network model that predicts workplace satisfaction of Primary/Elementary teachers as they vary between ± 3 standard deviations of the mean of the graphed trait (with other traits kept at average values).

**FIGURE 4:**
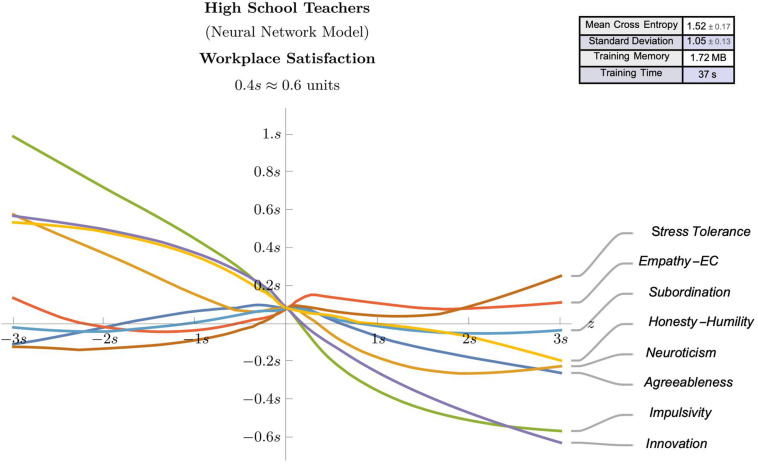
A (feed-forward) Neural Network model that predicts the work satisfaction of High School teachers as they vary between ± 3 standard deviations of the mean of the graphed trait (with other traits kept at average values).

**FIGURE 5:**
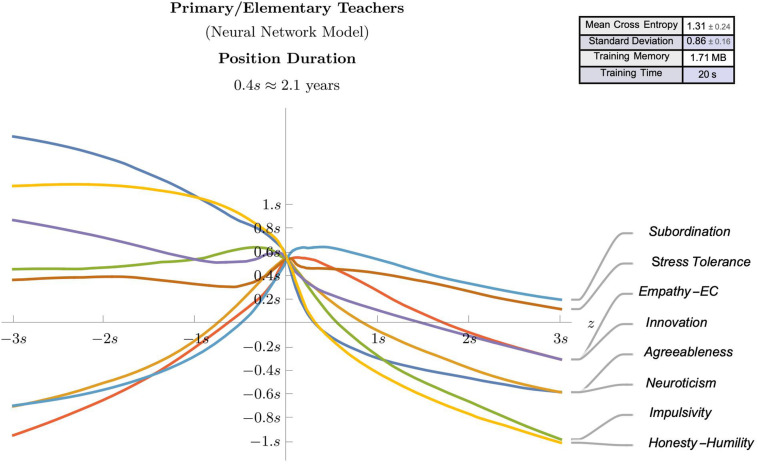
A (feed-forward) Neural Network model that predicts the position duration of Primary/Elementary teachers as they vary between ± 3 standard deviations of the mean of the graphed trait (with other traits kept at average values).

**FIGURE 6:**
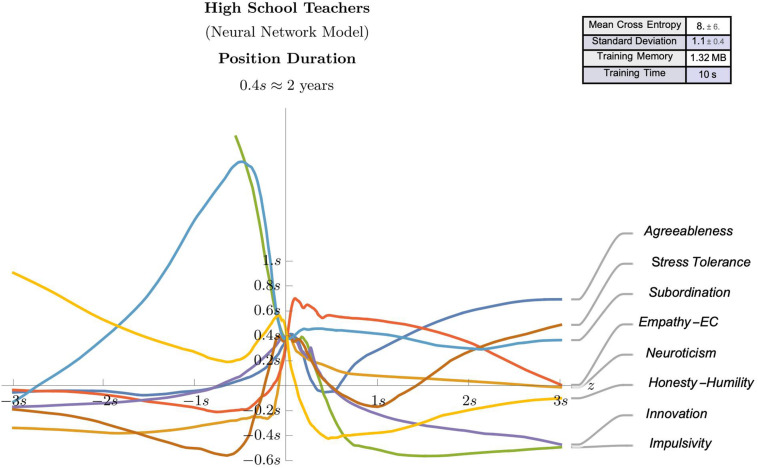
A (feed-forward) Neural Network model that predicts the position duration of High School teachers as they vary between ± 3 standard deviations of the mean of the graphed trait (with other traits kept at average values).

The observed effect sizes highlighted in the regression analyses, in combination with the theoretical framework offered some meaningful guidance regarding the potential predictive value of some selected psychological attributes (e.g., agreeableness, neuroticism, impulsivity, empathic concern, innovation, stress tolerance, subordination, and honesty-humility). Accordingly, these identified attributes were used as features in neural network models for suggesting predictive models for workplace satisfaction (Figures 3, 4) and position duration (Figures 5, 6) at the two different school levels. These (feed-forward) NN models allow predictions for teachers as they vary between ± 3 standard deviations of the mean of the graphed trait (with other traits kept at average values).

We use these models to compare and contrast the linear effects observed in the regression analyses. For instance, as can be seen in Figures 3–5, there is evidence of uniform linearity, however, uniform linearity is largely absent in Figure 6. Note that Figure 6 yielded a relatively large mean cross entropy of eight, which indicates that the predictive quality of the model predicting position duration for high school teachers is likely to be considerably lower than the predictive quality of the other three models (with respective cross entropies of 1:40, 1:52 and 1:31). While all models exhibited similar levels of fit, we will restrict our interpretation to the three former figures.

As can be seen in Figure 3, notable linear positive predictive effects were observed for stress tolerance (for workplace satisfaction) in primary/elementary teachers, however, in Figure 5, positive linear effects for subordination, empathic concern and neuroticism appear restricted to those scoring below average, with these effects diminishing or changing direction (neuroticism) for those above average. By contrast, in Figure 4, notable linear negative predictive effects are observed for impulsivity (for workplace satisfaction) and innovation (for position duration) in high school teachers. In Figure 5, the negative association between agreeableness and position duration for primary/elementary teachers that was observed in the regression analyses was also seen in the neural models albeit to a less pronounced degree. The role played by agreeableness in workplace satisfaction, however, (Figures 3, 4), exemplifies an asymmetric non-linearity whereby above average levels of agreeableness similarly predict lower workplace satisfaction in both teacher groups but below-average levels of agreeableness seem to have little predictive value. Also in Figure 5. subordination, empathic concern and neuroticism in primary/elementary teachers also all exhibited a striking asymmetry where the further a teacher was from the mean on the respective measure, the lower the position duration. While the regression analysis suggested a strong, negative association between impulsivity and position duration in primary/elementary teachers, this effect only held for above-average levels whereas below-average levels of impulsivity had very little predictive impact on position duration for this cohort (Figure 5). In contrast to these range of departures from linearity, both innovation and stress tolerance were relatively consistent linear predictors across teaching levels for both workplace satisfaction and position duration.

## Discussion

The aim of the present study was to measure theoretically and empirically valuable psychological attributes in an international sample of school teachers to determine the most valuable correlates of workplace satisfaction and position duration. Accordingly, we proposed 20 possible associations. However, we only found evidence for four of these. Specifically, we found that neuroticism negatively predicted workplace satisfaction in high school teachers (H2a), stress tolerance and organizational commitment positively predicted position duration in high school teachers (H3d and H3e), and impulsivity negatively predicted position duration in primary/elementary school teachers (H4b). We also found several unpredicted associations. Overall, agreeableness negatively predicted position duration. For primary/elementary teachers, empathic concern negatively predicted workplace satisfaction. For high school teachers, perspective taking negatively predicted position duration. The implications of these predicted and unpredicted associations are discussed below.

### Associations With Workplace Satisfaction

There were no notable predictors of workplace satisfaction for the entire sample according to the regression analyses. However, separate regressions for each school level revealed that neuroticism was negatively associated with workplace satisfaction for high school teachers, and empathic concern was negatively associated with workplace satisfaction for primary/elementary school teachers. Given that neuroticism is characterized by impatience, nervousness and insecurity (McCrae and Costa, 1987; Bastian et al., 2017), these traits potentially prevent high school teachers from achieving satisfaction in the workplace. For instance, difficulties in feeling secure and calm would undermine self-efficacy for achieving fulfilment whilst also enhancing a perception that their needs are not being met by the job or employer (Carless, 2005). Our findings are consistent with prior meta-analytic evidence showing that negative affectivity and neuroticism are associated with lower satisfaction in an organizational context (Judge et al., 2002). Sensitivity to negative affect may also explain the negative association between empathic concern and workplace satisfaction in primary/elementary teachers. For instance, a tendency to feel concern for others who may be experiencing negative affect (or are indeed highly neurotic) could also interfere with one’s own perception of satisfaction in the workplace. While speculative, this raises the possibility that highly neurotic individuals may infect sensitive others through their mood, thus broadly influencing workplace satisfaction.

### Associations With Position Duration

Agreeableness yielded the strongest association with position duration in the current study. Specifically, those higher in agreeableness (more people orientated) were predicted to leave positions sooner. Separate regression and neural analyses for each school level revealed that this negative association was driven largely by primary/elementary school teachers. Given that agreeableness is characterized by selflessness, flexibility and cooperation (McCrae and Costa, 1987; Barrick and Mount, 1991), these traits potentially allow primary/elementary teachers to shift more easily between positions and employers. Whilst agreeableness is an inherently desirable trait for employers to seek in a prospective teacher, the evidence presented here suggests that it may carry the risk of a shorter appointment. The negative association between agreeableness and position duration observed in the present study is somewhat inconsistent with prior research in other industries finding that those higher in agreeableness are less likely to turnover or intend to quit (Zimmerman, 2008; Swider and Zimmerman, 2010; Sarwar et al., 2013). As such, shorter position duration for those higher in agreeableness could reflect a greater capability to move fluidly between positions within or between schools.

Further meaningful effects were only observed when performing analyses on separate school levels. For primary/elementary teachers, impulsivity negatively associated with position duration, while organizational commitment positively associated with position duration in both teacher groups. Evidence that primary/elementary school teachers with higher impulsivity are more likely to change positions is consistent with prior literature that impulsivity is linked with turnover intentions (Allen et al., 2005) due to affect-driven quitting without fully evaluating the consequences (Holtom et al., 2008; Zimmerman, 2008; Hom et al., 2012). However, in our study, the neural network models suggest that this effect is only true for those with particularly high levels of impulsivity (scoring above the mean). Consistent with prior evidence that teachers with higher organizational commitment are less likely to quit (Conley and You, 2009), we found that organizational commitment predicted longer position duration in both teacher groups. Our results further support a wide body of literature recognizing organizational commitment as one of the leading correlates of organizational outcomes (Griffeth et al., 2000; Meyer et al., 2002; Saks Alan, 2006; Srivastava, 2013).

For high school teachers, stress tolerance positively associated with position duration, and perspective taking negatively associated with position duration. The stress tolerance effect is consistent with several studies reporting that high school teachers are vulnerable to stress and burnout (Johnson et al., 2005; Chaplain, 2008; von der Embse et al., 2016; Skaalvik and Skaalvik, 2017). While high school teachers and primary/elementary school teachers reported similar *levels* of stress tolerance, the regression analyses suggest that experiencing stress may be more problematic for high school teachers than it is for primary/elementary school teachers. That is, the evidence suggests that high school teachers are more likely to vacate a position if they have low stress tolerance, whereas stress tolerance was not interpreted to be associated with position duration for primary/elementary school teachers in the regression analyses. The neural network models help differentiate these divergent effects for each teaching group. Specifically, the positive association suggested by the regression analysis in high school teacher is most relevant to those with particularly high stress tolerance, whereas, for primary/elementary school teachers, the effect is actually the opposite for those with particularly high stress tolerance. That is, primary/elementary school teachers with higher levels of stress tolerance may be more likely to have shorter position durations.

The finding that perspective taking negatively predicts position duration in high school teachers is interesting given the importance placed upon social-emotional competencies in teaching contexts (Jennings and Greenberg, 2009; Jones and Bouffard, 2012). Indeed the evidence presented here suggests that teachers with low emotional awareness persist in their positions which emphasizes the importance of SEL training programs (Schonert-Reichl, 2017). The differential findings for empathic concern and perspective taking in the present study also emphasize the importance of separately measuring emotional and cognitive components of empathy as they appear to offer different utility in understanding both workplace satisfaction and position duration in teachers.

### Interpretation of Neural Network Models

The regression analyses are necessarily limited by their assumption of linearity when identifying explanatory variables in a given phenomenon and their subsequent incorporation into a suggestive predictive model. As such, we have attempted to balance these interpretations by employing complementary statistical techniques which may offer important theoretical and practical benefits. Indeed, these neural network models revealed some interesting patterns which deviate from linearity in this context, some of which have already been described. Some broader observations can also be made which highlight the discrepancies between these contrasting analytical approaches. For instance, linearity seems most justified when measuring satisfaction and least justified when measuring position duration, with a marked departure from linearity observed for predictors of position duration in high school teachers. While these models do not necessarily contradict the regression analyses, they instead offer a finer slicing of the data so that agreement with linear analyses can be confirmed. Importantly though, these neural network models are capable of determining when disagreements suggest more complex effects and the need for more data in corresponding subpopulations. By way of an example, while the agreeableness dimension was identified as a meaningful predictor in the regression analyses, the neural models indicated that its effect was more nuanced, depending on whether the teacher was above or below average on this dimension’s continuum. In some ways, this is unsurprising given there does not seem to be any *a priori* reason why the same deficit or surfeit in a trait should predict proportionally and yet, this is what they are constrained to do in models resting on linearity assumptions.

A further example of a novel complex effect relates to the honesty-humility dimension, which was not identified as a meaningful predictor in the regression analysis and yet showed a salient effect for position duration in primary/elementary teachers. That is, people who are presumed to be sincere, fair, and modest were more likely to have shorter appointments. Given that prior research has shown positive correlations between honesty-humility and tenure (Wiltshire et al., 2014; Ceschi et al., 2016), our findings suggest that these effects might be unique to the teaching context, or possibly not consistently linear in other contexts as well. Note that in the NN models, given the cross-sectional data slices, the predictive quality of the models is contingent on the stability of psychological traits over time, an assumption which, however, is widely indicated (Hampson and Goldberg, 2006). In fact, the literature review cataloged a range of conflicting predictive effects in the workplace that can perhaps be teased apart through the use of these highly predictive, neural network models.

### Theoretical and Practical Considerations

From a theoretical perspective, the present findings suggest that many of our social-emotional and motivational assumptions regarding teachers were not broadly supported, at least in this particular sample. However, consistent with some of the theoretical and empirical literature, we did find further evidence for key psychological factors which can be linked to markers of a successful placement. Specifically, neuroticism, empathy, stress tolerance, organizational commitment, and impulsivity were associated with either workplace satisfaction and/or position duration. Consistent with the SEL framework, teachers who exhibit maladaptive socio-emotional traits, such as neuroticism are less likely to experience workplace satisfaction (Jennings and Greenberg, 2009). That is, a tendency to negatively appraise events and situations, as a well as exhibiting insecurity would likely interfere with the capacity to achieve workplace satisfaction. While counterintuitive to the assumptions of the SEL framework, empathic concern and perspective taking might also be disruptive to achieving workplace satisfaction and position longevity. For instance, a person who is susceptible to experiencing and perceiving the emotions of others may also be especially sensitive to interpersonal negative affect. Thus, individuals high in both neuroticism and/or empathy may be at risk of diminished self-efficacy and increased burnout as a consequence of these negative emotional and cognitive states, further preventing them from achieving workplace satisfaction (Maslach et al., 2001). For position duration, we found evidence that stress tolerance and organization commitment might be protective factors while impulsivity might be a risk factor for teachers (and employers) who value position longevity. In line with the SEL competency of self-management, we have found evidence that the identification and management of stress is important for position longevity in high school teachers (CASEL, 2020). Furthermore, and consistent with our assumptions that international teachers may be sensitive to job security, the evidence here suggests that adopting an organizationally committed attitude might be one strategy for ensuring a longer appointment via an organizational allegiance. By contrast, and consistent with a wide body of theoretical and empirical literature, those high in impulsivity may have shorter appointments because their hypersensitivity to novelty and reward encourages them to continually pursue different working conditions (Wood et al., 2011; Krupić and Corr, 2020). This is also consistent with evidence that international school teachers are often motivated by the opportunity to travel and experience new places (Jennings and Greenberg, 2009; Savva, 2015).

From a recruitment perspective, the current study raises several considerations. Firstly, the evidence presented here suggests that workplace satisfaction in teachers does not readily associate with psychological attributes, which is consistent with some prior studies on job satisfaction (Thomas et al., 2004). Thus, it is possible that organizational attributes are more important for predicting (or possibly moderating the association between) psychological attributes and workplace satisfaction. For instance, several studies have found that factors such as management style, work environment and promotion opportunities are important for occupational satisfaction (Astrauskaite et al., 2011; Bogler and Nir, 2014; Knapp et al., 2017). Secondly, we found evidence that high school teachers who are emotionally unstable are less likely to be satisfied in the workplace, a common observation in other industries (Connolly and Viswesvaran, 2000; Judge et al., 2001, 2002). Therefore, employers of high school teachers may find the assessment of emotional stability helpful for avoiding the likelihood of dissatisfied employees, which could have a broader adverse impact on organizational culture. Screening for emotional stability may be particularly valuable when recruiting for more demanding work contexts where resources may be limited.

Similar to workplace satisfaction, relatively few psychological attributes were associated with position duration. However, teachers high in desirable attributes such as agreeableness and perspective taking also reported shorter appointments. An important implication of these associations is that employers who seek teachers with highly desirable traits may also be at greater risk of these teachers changing positions more quickly. That is, while socially and emotionally competent individuals may enrich an organization, they may also be more flexible, fluid and in demand with respect to other employment opportunities. Indeed, the evidence here raises some considerations when making recruitment decisions, which are outlined below.

Teachers with highly employable traits are likely to be more capable of movement within and between organizations because they are more comfortable with the prospect of integrating into a new work environment. Employers should carefully examine the context in which a position is offered to determine the potential impact of employing teachers who are socially competent. For instance, if there is little opportunity for promotion, then highly employable individuals may vacate positions more quickly to take a position at another organization. This notion is supported by research finding a positive correlation between available alternatives and turnover (Swider et al., 2011). However, if other attractive positions are available within the same organization then employers may be able to more readily retain these teachers. Given that some positions are only offered on a short to medium term basis, seeking highly flexible teachers could be advantageous, particularly in the international schooling sector. If longer position duration is the objective, then employers could look to balance agreeableness and perspective-taking with other traits which correlated with longer appointments, such as organizational commitment in high school teachers, or low impulsivity in primary/elementary school teachers.

The results of the current study further support the importance of the fit between a teacher and a particular position to increase the likelihood of satisfaction and reduce the likelihood of premature turnover (Ellis et al., 2017). Given that the correlates in our study associated differently across school levels (high school vs. primary/elementary), we assume that varying combinations of psychological and organizational factors are important for making recruitment decisions in different contexts. These considerations should extend beyond the correlates we have identified as meaningful in our study because certain teaching areas (e.g., visual arts and physical education) may demand specific combinations of traits (e.g., extraversion and resilience). Employers should also consider the possibility of an optimal tenure for a given position. For instance, some positions might provide suitable training grounds where teachers can develop skills and confidence before moving into other positions or organizations. It is also plausible that remaining in the same position in the same organization for too long could lead to apathy and loss of motivation, which can be counterproductive (Hom, 2012). Redeployment, promotion or turnover can be essential for refreshing motivation and accountability for the quality of one’s work. Indeed, it can be argued that retention of an employee is not always an effective outcome for an organization (Boswell et al., 2017), particularly when low performing employees do not regularly vacate positions (Ingersoll, 2001). That is, long position duration could also be a marker of elusive “deadwood” (Ference et al., 1977), or poor caliber employees who have low employment potential, evidenced by a lack of alternative employment opportunities.

One notable finding of the present study was the absence of an association between satisfaction and position duration, which is consistent with several studies (Boswell, 2006; Furnham, 2009; Lizano and Mor Barak, 2015; Boswell et al., 2017). Our data further demonstrates that exploring these as discrete organizational outcomes was justifiable. The data also supports the argument that leaving a position is only one strategy for resolving dissatisfaction (Rosse and Hulin, 1985; Hom et al., 2017). That is, our study suggests that, for teachers with high workplace satisfaction, they are no more likely to continue in a position than teachers with low workplace satisfaction. In practical terms, schools should not expect that efforts to enhance workplace satisfaction will necessarily translate into higher retention rates.

Given the paucity of research connecting psychological traits to international school placements, our focus was largely on theoretical, explanatory goals but there remains, however, the over-arching practical imperative to deploy predictive models which aim to match a dimension-rich individual to the ethos of a given school. For example, regression analyses suggest that within the primary/elementary context an “all other factors being equal” unit increase in agreeableness can decrease tenure by a somewhat specific 3.53 years. How such an interpretation should be combined into a practical application is not so straightforward because the selection of a candidate may be complicated by school demands and when candidates score similarly on some dimensions but inevitably vary across others. One approach was to build a neural network model that offers significant advantages for more precisely predicting workplace satisfaction and position duration. Although computationally more intensive and often requiring larger data sets, the use of so-called regularization^2^ in the generation of machine-learned models provides a rigorous, information-theoretical approach toward minimizing the risks of over-fitting (MacKay, 2005; Goodfellow et al., 2016; Yarkoni and Westfall, 2017). This contrasts with linear hierarchical regression which, while more tractable and explanatory, proceeds by systematically improving model fits at each variable-adding step (Cohen et al., 2003). While the extra complexity of machine-learned can certainly reduce their explanatory power, their extra predictive power positions them as inferentially valuable for detecting true effects (Guyon et al., 2010; Orrù et al., 2020). While the data sets used in this analysis are small for such advantages, we have nonetheless take some preliminary steps in this methodological direction.

### Limitations and Future Directions

There were several limitations that constrain the conclusions that can be drawn from the present study. However, these limitations can be positioned as valuable considerations for future efforts in this area. Firstly, our measurement of workplace satisfaction and position duration should be interpreted differently to popular measures of job satisfaction and turnover intentions. For instance, the attributes which associate with job satisfaction may be meaningfully different to those that associate with workplace satisfaction. This study has also focused on individual differences rather than organizational factors for understanding satisfaction and position duration. Ingersoll (2001) would argue that organizational characteristics are equally important in understanding staffing challenges in the teaching profession. Our data support this conclusion because there were relatively few meaningful psychological correlates emerging for workplace satisfaction and position duration. As we were unable to report on performance outcomes or organizational citizenship behaviors, which are valuable markers for organizational functioning (Judge et al., 2017), our conclusions are necessarily limited regarding placement success. Securing a data set of individual differences matched with organizationally provided data on performance is a more challenging research design but one that we encourage other researchers to pursue in the international education industry. Collecting contextual and cultural data (school size, socioeconomic status, and student characteristics etc.) could also be important for understanding satisfaction, retention and the mixed findings across school levels. Our sample was limited due to an over-representation of certain regions (e.g., Asia and the Middle East) and under-representation of others (e.g., North America and Europe), whilst also consisting of teachers in international schools. While we expect some of our findings may broadly apply to teachers, international schools are a unique context and, as such, we caution against generalizing our findings beyond the school type and regions that we surveyed. With greater representation across different regions and school types, it would be worthwhile examining these contexts separately to determine if different correlates emerge.

Our method of data collection was also disadvantaged by a reliance on self-report and a relatively long completion time (median of 35 min). Note that we randomized the order of the instruments to mitigate any systematic fatigue and we collected social desirability responses in an attempt to control for possible response bias in our data. Also note that social desirability bias may have been minimized because the data collection was propositioned for research rather than a recruitment screening. Those in recruitment contexts should be mindful of a greater likelihood of socially desirable responding if these instruments are used for profiling teaching candidates. Despite our efforts to manage response bias and fatigue, we suggest that future efforts avoid a reliance on online self-report and reduce the administration length by focusing on the correlates that are most likely to be valuable for a relevant context. We also recognize that several of our chosen instruments yielded low internal consistency (alpha) estimates, notably conscientiousness (0.65), neuroticism (0.64), and honest-humility (0.63). While these estimates could be unique to our sample, we suggest that future researchers opt to use longer forms of these instruments to reduce possible measurement error.

In this study we also instigated a framework and progression for detecting non-linear, interactive effects through the introduction of neural networks to model outcomes based on psychometric attributes. However, a clear limitation in the presented neural network models is the relatively small sample size. To claim firmer footing for the observed effects, larger cohorts and, in turn, more capable computational resources are required. Another limitation with respect to the neural models is that the produced graphs have been isolated according to when other personality dimensions are set to average values. To properly understand potential mixed effects of multiple personality dimensions, a more thorough investigation is needed, perhaps best facilitated through the use of interactive technologies.

Despite these limitations, our study does have several strengths. Most notably, very little quantitative work has been done to understand the role of psychological attributes in the successful placement of international teachers, so our research provides a valuable descriptive data set, and suggested models, useful for further studies into establishing important psychological predictors. A further advantage of our design is that the instruments selected were freely available and relatively brief. Thus, our methodology is well-suited to repeated measurement and for use by the non-academic sector. This is particularly important for research and recruitment applications in the future because some individual and workplace factors can fluctuate quickly, therefore longitudinal work is recommended to allow a more dynamic, rather than static, assessment of psychological attributes. We also regard our use of complementary use of machine-learned models which do not depend on model-theoretical assumptions around linearity, multi-collinearity and homoscedasticity as a key strength. Such models have increasing applicability in psychometrics and more generally in psychology as larger data sets open up opportunities to model underlying cognitive processes instead of having to rely on hypothesized statistical distributions (Yarkoni and Westfall, 2017; Orrù et al., 2020). The advantage of neural networks here is that the inherent cross-validation (here using Mathematica’s Predict functionality) can detect a level of probable cause-effect, which can establish the methodology’s viability and potential for larger datasets. Hence we see the advantage of this dual, sequenced approach whereby main effects are detected in initial, linear approximations before being incorporated into predictive models capable of revealing more complex effects that are potentially more applicable (Urban and Gates, 2019). We hope our example encourages others to pursue a similar process when attempting to understand and identify meaningful patterns of prediction.

### Recommendations and Conclusions

In a profession characterized by high levels of turnover, it is helpful to make some recommendations for improving retention in teachers. Note that the nature of the teaching position is likely to be imperative when deciding which psychological attributes are sought in a teacher. Therefore, employers should carefully analyze a role before seeking a potential teacher. Important considerations include the duration of the contract, the school level and whether there are promotion opportunities within the school. When seeking teachers for short- to medium-term contracts (e.g., <5 years) with little opportunity for continued employment in another position, we can recommend candidates who score high in agreeableness. For primary/elementary school teachers, a combination of low impulsivity and high organizational commitment is particularly desirable for maximizing position duration. For high school teachers, a combination of high stress tolerance and organizational commitment is particularly desirable for maximizing position duration. Furthermore, and in line with prior research, we recommend avoiding high levels of neuroticism to increase the likelihood of workplace satisfaction.

In conclusion, the current study demonstrated that individual differences, particularly personality traits, continue to be a valuable correlates of employee outcomes. We also report that correlates do not perform consistently across school levels, further supporting the understanding that correlates of organizational outcomes are likely to be industry and role specific. This calls for regular measurement of psychological attributes and organizational outcomes in teachers as well as further efforts to determine which combinations of attributes best predict successful placement for particular teaching contexts.

## Data Availability Statement

The raw data supporting the conclusions of this article will be made available by the corresponding author, by request, without undue reservation.

## Ethics Statement

The data collection procedures involving human participants were reviewed and approved by the Edith Cowan University Human Research Ethics Committee. The participants provided informed online consent to participate in this study.

## Author Contributions

RH prepared the manuscript, devised the survey battery and data collection, performed the data analysis, and interpreted the results as well as engaged with reviewer feedback. MM provided the leadership to initiate the study, secured funding, and provided ongoing feedback on the manuscript’s direction. RM contributed to the study’s design and manuscript’s overall narrative, particularly in relation to the Neural Network data-analysis. All authors contributed to the study’s article and approved the submitted version.

## Conflict of Interest

RM was employed by company OnSoc. The remaining authors declare that the research was conducted in the absence of any commercial or financial relationships that could be construed as a potential conflict of interest.

## Footnotes

1 For readers who wish to draw parallels between effect size estimates and traditional significance thresholds (*p* < 0.05), typical to large effect sizes in a sample as large as the one reported in this study are all comfortably significant. Furthermore, significance at *p* < 0.05 can also be assumed when 95% confidence intervals do not overlap with zero. We indicate typical to large effect sizes and report 95% confidence intervals in our results section.

2 Also known variously as *cross-validation*, or employing the *bias-variance tradeoff, principle of parsimony, or Ockham’s razor.*

## References

[B1] Agarwal U. A. Gupta V. (2018). Relationships between job characteristics, work engagement, conscientiousness and managers’ turnover intentions: a moderated-mediation analysis. *Pers. Rev.* 47 353–377. 10.1108/PR-09-2016-0229

[B2] Allen D. G. Bryant P. C. Vardaman J. M. (2010). Retaining talent: replacing misconceptions with evidence-based strategies. *Acad. Manag. Perspect.* 24 48–64. 10.5465/amp.24.2.48

[B3] Allen D. G. Weeks K. P. Moffitt K. R. (2005). Turnover intentions and voluntary turnover: the moderating roles of self-monitoring, locus of control, proactive personality, and risk aversion. *J. Appl. Psychol.* 90 980–990. 10.1037/0021-9010.90.5.980 16162070

[B4] Allen N. J. Meyer J. P. (1990). The measurement and antecedents of affective, continuance and normative commitment to the organization. *J. Occup. Psychol.* 63 1–18. 10.1111/j.2044-8325.1990.tb00506.x

[B5] Ashton M. C. Lee K. (2005). Honesty-humility, the big five, and the five-factor model. *J. Personal.* 73 1321–1354. 10.1111/j.1467-6494.2005.00351.x 16138875

[B6] Ashton M. C. Lee K. (2009). The HEXACO-60: a short measure of the major dimensions of personality. *J. Pers. Assess.* 91 340–345. 10.1080/00223890902935878 20017063

[B7] Ashton M. C. Lee K. de Vries R. E. (2014). The hexaco honesty-humility, agreeableness, and emotionality factors: a review of research and theory. *Personal. Soc. Psychol. Rev.* 18 139–152. 10.1177/1088868314523838 24577101

[B8] Astrauskaite M. Vaitkevicius R. Perminas A. (2011). Job satisfaction survey: a confirmatory factor analysis based on secondary school teachers’ sample. *Int. J. E Bus. Manag.* 6:41.

[B9] Australian Institute for Teaching and School Leadership (2016). *Spotlight: What do we Know About Early Career Teacher Attrition Rates in Australia?.* Melbourne: Australian Institute for Teaching and School Leadership.

[B10] Avery R. E. Smillie L. D. Fife-Schaw C. R. (2015). Employee achievement orientations and personality as predictors of job satisfaction facets. *Personal. Individ. Differ.* 76 56–61. 10.1016/j.paid.2014.11.037

[B11] Avey J. B. Luthans F. Jensen S. M. (2009). Psychological capital: a positive resource for combating employee stress and turnover. *Hum. Resou. Manag.* 48 677–693. 10.1002/hrm.20294

[B12] Bailey L. (2015). Reskilled and ‘running ahead’: teachers in an international school talk about their work. *J. Res. Int. Educ.* 14 3–15. 10.1177/1475240915572949

[B13] Bandura A. (1977). Self-efficacy: toward a unifying theory of behavioral change. *Psychol. Rev.* 84 191–215. 847061 10.1037//0033-295x.84.2.191

[B14] Barrick M. R. Mount M. K. (1991). The big five personality dimensions and job performance: a meta-analysis. *Pers. Psychol.* 44 1–26. 10.1111/j.1744-6570.1991.tb00688.x

[B15] Barrick M. R. Stewart G. L. Piotrowski M. (2002). Personality and job performance: test of the mediating effects of motivation among sales representatives. *J. Appl. Psychol.* 87 43–51. 10.1037/0021-9010.87.1.43 11916215

[B16] Bastian K. C. McCord D. M. Marks J. T. Carpenter D. (2017). A temperament for teaching? Associations between personality traits and beginning teacher performance and retention. *AERA Open* 3:2332858416684764 10.1177/2332858416684764

[B17] Beltman S. Mansfield C. Price A. (2011). Thriving not just surviving: a review of research on teacher resilience. *Educ. Res. Rev.* 6 185–207. 10.1016/j.edurev.2011.09.001

[B18] Blase J. J. (1982). A social-psychological grounded theory of teacher stress and burnout. *Educ. Adm. Q.* 18 93–113. 10.1177/0013161X82018004008

[B19] Bogler R. Nir A. E. (2014). The contribution of perceived fit between job demands and abilities to teachers’ commitment and job satisfaction. *Educ. Manag. Adm. Leadersh.* 43 541–560. 10.1177/1741143214535736

[B20] Bos N. Groeneveld C. van Bruggen J. Brand-Gruwel S. (2016). The use of recorded lectures in education and the impact on lecture attendance and exam performance. *Br. J. Educ. Technol.* 47 906–917. 10.1111/bjet.12300

[B21] Boswell W. (2006). Aligning employees with the organization’s strategic objectives: out of ‘line of sight’, out of mind. *Int. J. Hum. Resour. Manag.* 17 1489–1511. 10.1080/09585190600878071

[B22] Boswell W. R. Gardner R. G. Wang J. (2017). Is retention necessarily a win? Outcomes of searching and staying. *J. Vocat. Behav.* 98 163–172. 10.1016/j.jvb.2016.11.006

[B23] Bowling N. A. Khazon S. Meyer R. D. Burrus C. J. (2015). Situational strength as a moderator of the relationship between job satisfaction and job performance: a meta-analytic examination. *J. Bus. Psychol.* 30 89–104. 10.1007/s10869-013-9340-7

[B24] Brannan M. J. (2015). ‘You’re not going anywhere’: employee retention, symbolic violence and the structuring of subordination in a uk-based call centre. *Sociol. Rev.* 63 801–819. 10.1111/1467-954X.12312

[B25] Bunnell T. (2016). Teachers in international schools: a global educational ‘precariat’? *Global., Soc. Educ.* 14 543–559.

[B26] Bunnell T. (2019^∗^). Leadership of ‘messy, tense international schools’: the potential scope for a fresh, positive lens of inquiry. *Int. J. Leadersh. Educ.* 1–13. 10.1080/13603124.2019.1690708

[B27] Burke L. A. Witt L. A. (2004). Personality and high-maintenance employee behavior [journal article]. *J. Bus. Psychol.* 18 349–363. 10.1023/b:Jobu.0000016711.90781.58

[B28] Cable D. M. DeRue D. S. (2002). The convergent and discriminant validity of subjective fit perceptions. *J. Appl. Psychol.* 87 875–884. 10.1037/0021-9010.87.5.875 12395812

[B29] Carless S. A. (2005). Person–job fit versus person–organization fit as predictors of organizational attraction and job acceptance intentions: a longitudinal study. *J. Occup. Organ. Psychol.* 78 411–429. 10.1348/096317905x25995

[B30] CASEL (2020). *CASEL’s SEL Framework: What are the Core Competence Areas and Where are They Promoted?.* Chicago, IL: CASEL.

[B31] Ceschi A. Sartori R. Dickert S. Costantini A. (2016). Grit or honesty-humility? New insights into the moderating role of personality between the health impairment process and counterproductive work behavior [Original Research]. *Front. Psychol.* 7:1799. 10.3389/fpsyg.2016.01799 28018250 PMC5147463

[B32] Chandler J. (2010). The role of location in the recruitment and retention of teachers in international schools. *J. Res. Int. Educ.* 9 214–226.

[B33] Chang M.-L. (2009). An appraisal perspective of teacher burnout: examining the emotional work of teachers. *Educ. Psychol. Rev.* 21 193–218. 10.1007/s10648-009-9106-y

[B34] Chaplain R. P. (2008). Stress and psychological distress among trainee secondary teachers in England. *Educ. Psychol.* 28 195–209. 10.1080/01443410701491858

[B35] Charbonnier-Voirin A. Roussel P. (2012). Adaptive performance: a new scale to measure individual performance in organizations. *Can. J. Adm. Sci. Rev. Can. Sci. Adm.* 29 280–293. 10.1002/cjas.232

[B36] Cohen J. Cohen P. West S. G. Aiken L. S. (2003). *Applied Multiple Regression/Correlation Analysis for the Behavioral Sciences*, 3rd Edn. New York: Routledge 10.4324/9780203774441

[B37] Collie R. J. (2012). School climate and socialemotional learning: predicting teacher stress, job satisfaction, and teaching efficacy. *J. Educ. Psychol.* 104: 1189.

[B38] Comber B. Nixon H. (2009). Teachers’ work and pedagogy in an era of accountability. *Discourse* 30 333–345. 10.1080/01596300903037069

[B39] Conley S. You S. (2009). Teacher role stress, satisfaction, commitment, and intentions to leave: a structural model. *Psychol. Rep.* 105(3 Pt 1), 771–786. 10.2466/pr0.105.3.771-786 20099538

[B40] Connolly J. J. Viswesvaran C. (2000). The role of affectivity in job satisfaction: a meta-analysis. *Personal. Individ. Differ.* 29 265–281. 10.1016/S0191-8869(99)00192-0

[B41] Coutu D. L. (2002). How resilience works. *Harv. Bus. Rev.* 80 46–50,52,55assim. 12024758

[B42] Crant J. M. (2000). Proactive behavior in organizations. *J. Manag.* 26 435–462.

[B43] Crede M. Harms P. Niehorster S. Gaye-Valentine A. (2012). An evaluation of the consequences of using short measures of the Big Five personality traits. *J. Pers. Soc. Psychol.* 102 874–888. 10.1037/a0027403 22352328

[B44] Cumming G. (2013). The new statistics: why and how. *Psychol. Sci.* 25 7–29. 10.1177/0956797613504966 24220629

[B45] Davis M. H. (1983). Measuring individual differences in empathy: evidence for a multidimensional approach. *J. Pers. Soc. Psychol.* 44 113–126. 10.1037/0022-3514.44.1.113

[B46] Deci E. L. Ryan R. M. (1985). *Intrinsic Motivation and Self-Determination in Human Behavior*. Boston, MA: Springer Available online at: 10.1007/978-1-4899-2271-7

[B47] de Vries R. E. van Gelder J.-L. (2015). Explaining workplace delinquency: the role of Honesty–Humility, ethical culture, and employee surveillance. *Personal. Individ. Differ.* 86 112–116. 10.1016/j.paid.2015.06.008

[B48] DeZoort T. Roskos-Ewoldsen D. R. (1997). The submissiveness to organizational authority scale as a measure of authoritarianism. *J. Soc. Behav. Personal.* 12 651–670.

[B49] Donnellan M. B. Oswald F. L. Baird B. M. Lucas R. E. (2006). The mini-IPIP scales: tiny-yet-effective measures of the big five factors of personality. *Psychol. Assess.* 18 192–203. 10.1037/1040-3590.18.2.192 16768595

[B50] Ellis C. Skidmore S. T. Combs J. P. (2017). The hiring process matters: the role of person–job and Person–Organization fit in teacher satisfaction. *Educ. Adm. Q.* 53 448–474. 10.1177/0013161X16687007

[B51] Ference T. P. Stoner J. A. Warren E. K. (1977). Managing the career plateau. *Acad. Manag. Rev.* 2 602–612. 10.2307/257512

[B52] Fuller B. Marler L. E. (2009). Change driven by nature: a meta-analytic review of the proactive personality literature. *J. Vocat. Behav.* 75 329–345. 10.1016/j.jvb.2009.05.008

[B53] Furnham A. (2009). Personality, motivation and job satisfaction: hertzberg meets the big five. *J. Manag. Psychol.* 24 765–779. 10.1108/02683940910996789

[B54] Furnham A. Petrides K. V. Jackson C. J. Cotter T. (2002). Do personality factors predict job satisfaction? *Personal. Individ. Differ.* 33 1325–1342.

[B55] Gignac G. E. Szodorai E. T. (2016). Effect size guidelines for individual differences researchers. *Personal. individ. Differ.* 102 74–78. 10.1016/j.paid.2016.06.069

[B56] Goodfellow I. Bengio Y. Courville A. Bengio Y. (2016). *Deep Learning*, Vol. 1 Cambridge: MIT press.

[B57] Griffeth R. W. Hom P. W. Gaertner S. (2000). A meta-analysis of antecedents and correlates of employee turnover: update, moderator tests, and research implications for the next millennium. *J. Manag.* 26 463–488.

[B58] Gu Q. Day C. (2007). Teachers resilience: a necessary condition for effectiveness. *Teach. Teach. Educ.* 23 1302–1316. 10.1016/j.tate.2006.06.006

[B59] Gu Q. Day C. (2013). Challenges to teacher resilience: conditions count. *Br. Educ. Res. J.* 39 22–44. 10.1080/01411926.2011.623152

[B60] Guyon I. Saffari A. Dror G. Cawley G. (2010). Model selection: beyond the bayesian/frequentist divide. *J. Mach. Learn. Res.* 11 61–87.

[B61] Hampson S. E. Goldberg L. R. (2006). A first large cohort study of personality trait stability over the 40 years between elementary school and midlife. *J. Pers. Soc. Psychol.* 91 763–779. 10.1037/0022-3514.91.4.763 17014298 PMC2247365

[B62] Hancock J. I. Allen D. G. Bosco F. A. McDaniel K. R. Pierce C. A. (2011). Meta-analytic review of employee turnover as a predictor of firm performance. *J. Manag.* 39 573–603. 10.1177/0149206311424943

[B63] Hanushek E. A. Rivkin S. G. Schiman J. C. (2016). Dynamic effects of teacher turnover on the quality of instruction. *Econ. Educ. Rev.* 55 132–148. 10.1016/j.econedurev.2016.08.004

[B64] Harms C. Pooley J. A. Cohen L. (2017). The protective factors for resilience scale (PFRS): development of the scale. *Cogent Psychol.* 4:1400415 10.1080/23311908.2017.1400415

[B65] Hart C. M. Ritchie T. D. Hepper E. G. Gebauer J. E. (2015). The balanced inventory of desirable responding short form (BIDR-16). *SAGE Open* 5:2158244015621113 10.1177/2158244015621113

[B66] Hayden M. Thompson J. J. (2008). *International Schools: Growth and Influence.* Paris: United Nations Educational, Scientific and Cultural Organization.

[B67] Holtom B. C. Mitchell T. R. Lee T. W. Eberly M. B. (2008). Turnover and retention research: a glance at the past, a closer review of the present, and a venture into the future. *Acad. Manag. Ann.* 2 231–274. 10.5465/19416520802211552

[B68] Hom P. W. (2012). Reviewing employee turnover: focusing on proximal withdrawal states and an expanded criterion. *Psychol. Bull.* 138 831–858. 22925138 10.1037/a0027983

[B69] Hom P. W. Kinicki A. J. (2001). Toward a greater understanding of how dissatisfaction drives employee turnover. *Acad. Manag. J.* 44 975–987. 10.5465/3069441 3069441

[B70] Hom P. W. Lee T. W. Shaw J. D. Hausknecht J. P. (2017). One hundred years of employee turnover theory and research. *J. Appl. Psychol.* 102 530–545. 28125259 10.1037/apl0000103

[B71] Hom P. W. Mitchell T. R. Lee T. W. Griffeth R. W. (2012). Reviewing employee turnover: focusing on proximal withdrawal states and an expanded criterion. *Psychol. Bull.* 138 831–858. 10.1037/a0027983 22925138

[B72] Hong J. Y. (2012). Why do some beginning teachers leave the school, and others stay? Understanding teacher resilience through psychological lenses. *Teach. Teach.* 18 417–440. 10.1080/13540602.2012.696044

[B73] Howard S. Johnson B. (2004). Resilient teachers: resisting stress and burnout [journal article]. *Soc. Psychol. Educ.* 7 399–420. 10.1007/s11218-004-0975-0

[B74] Hudgins T. A. (2016). Resilience, job satisfaction and anticipated turnover in nurse leaders. *J. Nurs. Manag.* 24 E62–E69. 10.1111/jonm.12289 25782613

[B75] Hughes G. D. (2012). Teacher retention: teacher characteristics, school characteristics, organizational characteristics, and teacher efficacy. *J. Educ. Res.* 105 245–255.

[B76] Hulpia H. Devos G. Rosseel Y. (2009). The relationship between the perception of distributed leadership in secondary schools and teachers’ and teacher leaders’ job satisfaction and organizational commitment. *Sch. Effect. Sch. Improv.* 20 291–317. 10.1080/09243450902909840

[B77] Ilies R. Fulmer I. S. Spitzmuller M. Johnson M. D. (2009). Personality and citizenship behavior: the mediating role of job satisfaction. *J. Appl. Psychol.* 94 945–959. 10.1037/a0013329 19594236

[B78] Ingersoll R. M. (2001). Teacher turnover and teacher shortages: an organizational analysis. *Am. Educ. Res. J.* 38 499–534.

[B79] Ingoglia S. Lo Coco A. Albiero P. (2016). Development of a brief form of the interpersonal reactivity index (B-IRI). *J. Pers. Assess.* 98 461–471. 10.1080/00223891.2016.1149858 27050826

[B80] Jennings P. A. Brown J. L. Frank J. L. Doyle S. Oh Y. Davis R. (2017). Impacts of the CARE for teachers program on teachers’ social and emotional competence and classroom interactions. *J. Educ. Psychol.* 109 1010–1028. 10.1037/edu0000187

[B81] Jennings P. A. Greenberg M. T. (2009). The prosocial classroom: teacher social and emotional competence in relation to student and classroom outcomes. *Rev. Educ. Res.* 79 491–525. 10.3102/0034654308325693

[B82] Johnson M. K. Rowatt W. C. Petrini L. (2011). A new trait on the market: honesty–Humility as a unique predictor of job performance ratings. *Personal. Individ. Differ.* 50 857–862. 10.1016/j.paid.2011.01.011

[B83] Johnson S. Cooper C. Cartwright S. Donald I. Taylor P. Millet C. (2005). The experience of work-related stress across occupations. *J. Manag. Psychol.* 20 178–187. 10.1108/02683940510579803

[B84] Jones S. M. Bouffard S. M. (2012). Social and emotional learning in schools: from programs to strategies and commentaries. *Soc. Policy Rep.* 26 1–33. 10.1002/j.2379-3988.2012.tb00073.x32226269

[B85] Jones S. M. Bouffard S. M. Weissbourd R. (2013). Educators’ social and emotional skills vital to learning. *Phi Delta Kappan* 94 62–65.

[B86] Joo B.-K. Hahn H.-J. Peterson S. L. (2015). Turnover intention: the effects of core self-evaluations, proactive personality, perceived organizational support, developmental feedback, and job complexity. *Hum. Resour. Dev. Int.* 18 116–130. 10.1080/13678868.2015.1026549

[B87] Joslin P. (2002). Teacher relocation: reflections in the context of international schools. *J. Res. Int. Educ.* 1 33–62. 10.1177/1475240902001001268

[B88] Judge T. A. Heller D. Mount M. K. (2002). Five-factor model of personality and job satisfaction: a meta-analysis. *J. Appl. Psychol.* 87 530–541. 12090610 10.1037/0021-9010.87.3.530

[B89] Judge T. A. Thoresen C. J. Bono J. E. Patton G. K. (2001). The job satisfaction–job performance relationship: a qualitative and quantitative review. *Psychol. Bull.* 127 376–407. 10.1037/0033-2909.127.3.376 11393302

[B90] Judge T. A. Weiss H. M. Kammeyer-Mueller J. D. Hulin C. L. (2017). Job attitudes, job satisfaction, and job affect: a century of continuity and of change. *J. Appl. Psychol.* 102 356–374. 10.1037/apl0000181 28125260

[B91] Kim H. Kao D. (2014). A meta-analysis of turnover intention predictors among U.S. child welfare workers. *Child. Youth Servic. Rev.* 47 214–223. 10.1016/j.childyouth.2014.09.015

[B92] Klassen R. M. Usher E. L. Bong M. (2010). Teachers’ collective efficacy, job satisfaction, and job stress in cross-cultural context. *J. Exp. Educ.* 78 464–486. 10.1080/00220970903292975

[B93] Knapp J. R. Smith B. R. Sprinkle T. A. (2017). Is It the Job or the Support? Examining structural and relational predictors of job satisfaction and turnover intention for nonprofit employees. *Nonprofit Vol. Sector Q.* 46 652–671. 10.1177/0899764016685859

[B94] Krupić D. Corr P. J. (2020). How reinforcement sensitivity theory relates to self-determination theory. *Personal. Individ. Differ.* 155:109705.

[B95] Kukla-Acevedo S. (2009). Leavers, movers, and stayers: the role of workplace conditions in teacher mobility decisions. *J. Educ. Res.* 102 443–452. 10.3200/JOER.102.6.443-452

[B96] Kusurkar R. A. Croiset G. Ten Cate T. J. (2011). Twelve tips to stimulate intrinsic motivation in students through autonomy-supportive classroom teaching derived from self-determination theory. *Med. Teach.* 33 978–982. 10.3109/0142159x.2011.599896 22225435

[B97] Kyriacou C. (2001). Teacher stress: directions for future research. *Educ. Rev.* 53 27–35. 10.1080/00131910120033628

[B98] Kyriacou C. Sutcliffe J. (1979). Teacher stress and satisfaction. *Educ. Res.* 21 89–96. 10.1080/0013188790210202

[B99] Lam S.-F. Cheng R. W.-Y. Ma W. Y. K. (2008). Teacher and student intrinsic motivation in project-based learning [journal article]. *Instr Sci.* 37 565–578. 10.1007/s11251-008-9070-9

[B100] Li M. Wang Z. Gao J. You X. (2017). Proactive personality and job satisfaction: The mediating effects of self-efficacy and work engagement in teachers [journal article]. *Curr. Psychol.* 36 48–55. 10.1007/s12144-015-9383-1

[B101] Li N. Liang J. Crant J. M. (2010). The role of proactive personality in job satisfaction and organizational citizenship behavior: a relational perspective. *J. Appl. Psychol.* 95 395–404. 10.1037/a0018079 20230079

[B102] Liao H. Chuang A. (2004). A multilevel investigation of factors influencing employee service performance and customer outcomes. *Acad. Manag. J.* 47 41–58. 10.5465/20159559 20159559

[B103] Lizano E. L. Mor Barak M. (2015). Job burnout and affective wellbeing: a longitudinal study of burnout and job satisfaction among public child welfare workers. *Child. Youth Servic. Rev.* 55 18–28. 10.1016/j.childyouth.2015.05.005

[B104] Locke E. A. (1969). What is job satisfaction? *Organ. Behav. Hum. Perform.* 4 309–336. 10.1016/0030-5073(69)90013-0

[B105] Luthans F. (2002). The need for and meaning of positive organizational behavior. *J. Organ. Behav.* 23 695–706. 10.1002/job.165

[B106] MacKay D. J. (2005). *Information Theory, Inference and Learning Algorithms.* Cambridge: Cambridge University Press.

[B107] Mancuso S. V. Roberts L. White G. P. (2010). Teacher retention in international schools: The key role of school leadership. *J. Res. Int, Educ.* 9 306–323.

[B108] Mansfield C. F. Beltman S. Price A. McConney A. (2012). “Don’t sweat the small stuff:” Understanding teacher resilience at the chalkface. *Teach. Teach. Educ.* 28 357–367. 10.1016/j.tate.2011.11.001

[B109] Martocchio J. J. Judge T. A. (1997). Relationship between conscientiousness and learning in employee training: mediating influences of self-deception and self-efficacy. *J. Appl. Psychol.* 82 764–773. 9337608 10.1037/0021-9010.82.5.764

[B110] Maslach C. Schaufeli W. B. Leiter M. P. (2001). Job burnout. *Annu. Rev. Psychol.* 52 397–422. 10.1146/annurev.psych.52.1.397 11148311

[B111] Mathieu C. Fabi B. Lacoursière R. Raymond L. (2016). The role of supervisory behavior, job satisfaction and organizational commitment on employee turnover. *J. Manag. Organ.* 22 113–129. 10.1017/jmo.2015.25

[B112] Matzler K. Renzl B. (2007). Personality traits, employee satisfaction and affective commitment. *Total Qual. Manag. Bus. Excel.* 18 589–598. 10.1080/14783360601061528

[B113] McCrae R. R. Costa P. T. (1987). Validation of the five-factor model of personality across instruments and observers. *J. Pers. Soc. Psychol.* 52 81–90. 10.1037/0022-3514.52.1.81 3820081

[B114] Meyer J. P. Stanley D. J. Herscovitch L. Topolnytsky L. (2002). Affective, continuance, and normative commitment to the organization: a meta-analysis of antecedents, correlates, and consequences. *J. Vocat. Behav.* 61 20–52. 10.1006/jvbe.2001.1842

[B115] Miguel Dos Santos L. (2019). Recruitment and retention of international school teachers in remote archipelagic countries: the fiji experience. *Educ. Sci.* 9:132 10.3390/educsci9020132

[B116] Montgomery C. Rupp A. A. (2005). A meta-analysis for exploring the diverse causes and effects of stress in teachers. *Can. J. Educ. /Rev. Can. Éduc.* 28 458–486.

[B117] Mount M. Ilies R. Johnson E. (2006). Relationship of personality traits and counterproductive work behaviors: the mediating effects of job satisfaction. *Pers. Psychol.* 59 591–622. 10.1111/j.1744-6570.2006.00048.x

[B118] Näring G. Vlerick P. Van de Ven B. (2012). Emotion work and emotional exhaustion in teachers: the job and individual perspective. *Educ. Stud.* 38 63–72. 10.1080/03055698.2011.567026

[B119] Niemiec C. P. Ryan R. M. (2009). Autonomy, competence, and relatedness in the classroom: Applying self-determination theory to educational practice. *Theory Res. Educ.* 7 133–144.

[B120] Noels K. A. Clément R. Pelletier L. G. (1999). Perceptions of teachers’ communicative style and students’ intrinsic and extrinsic motivation. *Modern Lang. J.* 83 23–34. 10.1111/0026-7902.00003

[B121] Odland G. Ruzicka M. (2009). An investigation into teacher turnover in international schools. *J. Res. Int. Educ.* 8 5–29.

[B122] Organ D. W. Lingl A. (1995). Personality, satisfaction, and organizational citizenship behavior. *J. Soc. Psychol.* 135 339–350. 10.1080/00224545.1995.9713963

[B123] Orrù G. Monaro M. Conversano C. Gemignani A. Sartori G. (2020). Machine learning in psychometrics and psychological research. *Front. Psychol.* 10:2970. 10.3389/fpsyg.2019.02970 PMC6966768 31998200

[B124] Patrick B. C. Hisley J. Kempler T. (2000). “What’s everybody so excited about?”: the effects of teacher enthusiasm on student intrinsic motivation and vitality. *J. Exp. Educ.* 68 217–236.

[B125] Patton J. H. Stanford M. S. Barratt E. S. (1995). Factor structure of the Barratt impulsiveness scale. *J. Clin. Psychol.* 51 768–774. 8778124 10.1002/1097-4679(199511)51:6<768::aid-jclp2270510607>3.0.co;2-1

[B126] Paulhus D. L. (1984). Two-component models of socially desirable responding. *J. Pers. Soc. Psychol.* 46 598–609. 10.1037/0022-3514.46.3.598

[B127] Paulhus D. L. (1991). “Measurement and control of response bias,” in *Measures of Personality and Social Psychological Attitudes*, eds Robinson J. P. Shaver P. R. Wrightsman L. S. (Cambridge, MA: Academic Press.), 17–59. 10.1016/B978-0-12-590241-0.50006-X

[B128] Pearson C. L. Moomaw W. (2005). The relationship between teacher autonomy and stress, work satisfaction, empowerment, and professionalism. *Educ. Res. Q.* 29 38–54.

[B129] Petersen L. E. Dietz J. (2008). Employment discrimination: Authority figures’ demographic preferences and followers’ affective organizational commitment. *J. Appl. Psychol.* 93 1287–1300. 19025248 10.1037/a0012867

[B130] Price H. E. (2011). Principal–teacher interactions: how affective relationships shape principal and teacher attitudes. *Educ. Adm. Q.* 48 39–85. 10.1177/0013161X11417126

[B131] Pulakos E. D. Arad S. Donovan M. A. Plamondon K. E. (2000). Adaptability in the workplace: development of a taxonomy of adaptive performance. *J. Appl. Psychol.* 85 612–624. 10.1037/0021-9010.85.4.612 10948805

[B132] Raza M. Y. Akhtar M. W. Husnain M. Akhtar M. S. (2015). The impact of intrinsic motivation on employee’s job satisfaction. *Manag. Organ. Stud.* 2 80–88.

[B133] Ronfeldt M. Loeb S. Wyckoff J. (2013). How teacher turnover harms student achievement. *Am. Educ. Res. J.* 50 4–36. 10.3102/0002831212463813

[B134] Rosse J. G. Hulin C. L. (1985). Adaptation to work: an analysis of employee health, withdrawal, and change. *Organ. Behav. Hum. Decis. Proces.* 36 324–347. 10.1016/0749-5978(85)90003-2 10275698

[B135] Rubenstein A. L. Eberly M. B. Lee T. W. Mitchell T. R. (2018). Surveying the forest: a meta-analysis, moderator investigation, and future-oriented discussion of the antecedents of voluntary employee turnover. *Pers. Psychol.* 71 23–65. 10.1111/peps.12226

[B136] Ryan R. M. Deci E. L. (2000). Intrinsic and extrinsic motivations: classic definitions and new directions. *Contempo. Educ. Psychol.* 25 54–67. 10.1006/ceps.1999.1020 10620381

[B137] Ryan S. V. von der Embse N. P. Pendergast L. L. Saeki E. Segool N. Schwing S. (2017). Leaving the teaching profession: the role of teacher stress and educational accountability policies on turnover intent. *Teach. Teach. Educ.* 66 1–11. 10.1016/j.tate.2017.03.016

[B138] Saks Alan M. (2006). Antecedents and consequences of employee engagement. *J. Manag. Psychol.* 21 600–619. 10.1108/02683940610690169

[B139] Salgado J. F. (2002). The big five personality dimensions and counterproductive behaviors. *Int. J. Select. Assess.* 10 117–125. 10.1111/1468-2389.00198

[B140] Santo L. Pohl S. Battistelli A. (2013). Empathy in the emotional interactions with patients. Is it positive for nurses too? *J. Nurs. Educ. Pract.* 4 74–81. 10.5430/jnep.v4n2p74

[B141] Sarwar A. Hameed S. Aftab H. (2013). Study to explore the impact of personality traits on employee turnover in public and private sector. *Middle East J. Sci. Res.* 16 1249–1254. 10.5829/idosi.mejsr.2013.16.09.12027

[B142] Sass Daniel A. (2011). Predicting teacher retention using stress and support variables. *J. Educ. Adm.* 49 200–215.

[B143] Savva M. (2015). Characteristics of the international educator and the strategic role of critical incidents. *J. Res, Int. Educ.* 14 16–28. 10.1177/1475240915570548

[B144] Schleicher D. J. Watt J. D. Greguras G. J. (2004). Reexamining the job satisfaction-performance relationship: the complexity of attitudes. *J. Appl. Psychol.* 89 165–177. 10.1037/0021-9010.89.1.165 14769129

[B145] Schonert-Reichl K. A. (2017). Social and emotional learning and teachers. *Future Child.* 27 137–155.

[B146] Schwarzer R. Hallum S. (2008). Perceived teacher self-efficacy as a predictor of job stress and burnout: mediation analyses. *Appl. Psychol.* 57(Suppl. 1) 152–171. 10.1111/j.1464-0597.2008.00359.x

[B147] Seibert S. E. (1999). Proactive personality and career success. *J. Appl. Psychol.* 84 416–427. 10380421 10.1037/0021-9010.84.3.416

[B148] Shah M. J. Akhtar G. Zafar H. Riaz A. (2012^∗^). Job satisfaction and motivation of teachers of public educational institutions. *Int. J. Bus. Soc. Sci.* 3 271–281.

[B149] Skaalvik E. M. Skaalvik S. (2017). Still motivated to teach? A study of school context variables, stress and job satisfaction among teachers in senior high school. *Soc. Psychol. Educ.* 20 15–37. 10.1007/s11218-016-9363-9

[B150] Spector P. E. Michaels C. E. (1986). Personality and employee withdrawal: effects of locus of control on turnover. *Psychol. Rep.* 59 63–66. 10.2466/pr0.1986.59.1.63

[B151] Srivastava S. (2013). Job satisfaction and organizational commitment relationship: effect of personality variables. *Vision* 17 159–167. 10.1177/0972262912483529

[B152] Steinberg L. Sharp C. Stanford M. S. Tharp A. T. (2013). New tricks for an old measure: the development of the barratt impulsiveness scale-brief (BIS-Brief). *Psychol. Assess.* 25 216–226. 10.1037/a0030550 23148649

[B153] Sutcher L. Darling-Hammond L. Carver-Thomas D. (2016). *A Coming Crisis in Teaching? Teacher Supply, Demand and Shortages in the U.S.* Palo Alto, CA: Learning Policy Institute.

[B154] Swider B. W. Boswell W. R. Zimmerman R. D. (2011). Examining the job search–turnover relationship: The role of embeddedness, job satisfaction, and available alternatives. *J. Appl. Psychol.* 96 432–441. 10.1037/a0021676 21142342

[B155] Swider B. W. Zimmerman R. D. (2010). Born to burnout: a meta-analytic path model of personality, job burnout, and work outcomes. *J. Vocat Behav.* 76 487–506.

[B156] Thomas A. Buboltz W. C. Winkelspecht C. S. (2004). Job characteristics and personality as predictors of job satisfaction. *Organ. Anal.* 12 205–219. 10.1108/eb028993

[B157] Timmerman T. A. (2006). Predicting turnover with broad and narrow personality traits. *Int. J. Select. Assess.* 14 392–399. 10.1111/j.1468-2389.2006.00361.x

[B158] Tremblay M. A. Blanchard C. M. Taylor S. Pelletier L. G. Villeneuve M. (2009). Work extrinsic and intrinsic motivation scale: its value for organizational psychology research. *Can. J. Behav. Sci. Rev. Can. Sci. Comp.* 41 213–226. 10.1037/a0015167

[B159] Urban C. Gates K. (2019). Deep learning: a primer for psychologists. *PsyArXiv* [Preprint]. 10.31234/osf.io/4q8na 33793268

[B160] van Daal T. Donche V. De Maeyer S. (2014). The impact of personality, goal orientation and self-efficacy on participation of high school teachers in learning activities in the workplace. *Vocat. Learn.* 7 21–40. 10.1007/s12186-013-9105-5

[B161] von der Embse N. P. Pendergast L. L. Segool N. Saeki E. Ryan S. (2016). The influence of test-based accountability policies on school climate and teacher stress across four states. *Teach. Teach. Educ.* 59 492–502. 10.1016/j.tate.2016.07.013

[B162] Wasserstein R. L. Schirm A. L. Lazar N. A. (2019). Moving to a world beyond “p<0.05”. *Am. Stat.* 73 1–19. 10.1080/00031305.2019.1583913

[B163] Williams C. A. (1989). Empathy and burnout in male and female helping professionals. *Res. Nurs. Health* 12 169–178. 10.1002/nur.4770120307 2727323

[B164] Wiltshire J. Bourdage J. S. Lee K. (2014). Honesty-humility and perceptions of organizational politics in predicting workplace outcomes [journal article]. *J. Bus. Psychol.* 29 235–251. 10.1007/s10869-013-9310-0

[B165] Wood A. Rijsdijk F. Asherson P. Kuntsi J. (2011). Inferring causation from cross-sectional data: examination of the causal relationship between hyperactivity–impulsivity and novelty seeking [Original Research]. *Front. Genet.* 2:6. 10.3389/fgene.2011.00006 22303305 PMC3268378

[B166] Yarkoni T. Westfall J. (2017). Choosing prediction over explanation in psychology: lessons from machine learning. *Perspect. Psychol. Sci.* 12 1100–1122. 10.1177/1745691617693393 28841086 PMC6603289

[B167] Youssef C. M. Luthans F. (2007). Positive organizational behavior in the workplace: the impact of hope, optimism, and resilience. *J. Manag.* 33 774–800. 10.1177/0149206307305562

[B168] Yu X. Wang P. Zhai X. Dai H. Yang Q. (2015). The effect of work stress on job burnout among teachers: the mediating role of self-efficacy [journal article]. *Soc. Indic. Res.* 122 701–708. 10.1007/s11205-014-0716-5

[B169] Zeinabadi H. (2010). Job satisfaction and organizational commitment as antecedents of organizational citizenship behavior (ocb) of teachers. *Proc. Soc. Behav. Sci*, 5 998–1003. 10.1016/j.sbspro.2010.07.225

[B170] Zettler I. Hilbig B. E. (2010). Honesty–humility and a person–situation interaction at work. *Eur. J. Personal.* 24 569–582. 10.1002/per.757

[B171] Zheng Z. Gangaram P. Xie H. Chua S. Ong S. B. C. Koh S. E. (2017). Job satisfaction and resilience in psychiatric nurses: a study at the Institute of Mental Health. Singapore. *Int. J. Ment. Health Nurs.* 26 612–619. 10.1111/inm.12286 28160378

[B172] Zimmerman R. D. (2008). Understanding the impact of personality traits on individuals’ turnover decisions: a meta-analytic path model. *Pers. Psychol.* 61 309–348. 10.1111/j.1744-6570.2008.00115.x

[B173] Zins J. E. Weissberg R. P. Wang M. C. Walberg H. J. (2004). *Building Academic Success on Social and Emotional Learning: What Does the Research Say?.* New York, NY: Teachers College Press.

